# Supplementary material for: Metal-organic framework and inorganic glass composites
Source: Nat Commun. 2020 Nov 16;11:5800. doi: 10.1038/s41467-020-19598-9 (PMC7669864; doi:10.1038/s41467-020-19598-9)
Supplement: Supplementary file 1 — Supplementary Information [file 41467_2020_19598_MOESM1_ESM.pdf]

Supplementary Information

Louis Longley,[1] Courtney Calahoo,[2] René Limbach,[2] Yang Xia[2], Joshua M. Tuffnell,[1] Adam F. Sapnik,[1] Michael F. Thorne,[1] Dean S. Keeble,[3] David A. Keen,[4] Lothar Wondraczek[2] and Thomas D. Bennett[1]*

[1]Department of Materials Science and Metallurgy, University of Cambridge, CB3 0FS, UK

[2]Otto Schott Institute of Materials Research, University of Jena, Fraunhoferstrasse 6, 07743 Jena, Germany

[3]Diamond Light Source Ltd., Diamond House, Harwell Campus, Didcot, Oxfordshire OX11 0DE UK

[4]ISIS Facility, Rutherford Appleton Laboratory, Harwell Campus, Didcot, Oxfordshire OX11 0QX UK

tdb35@cam.ac.uk

Supplementary Methods

Differential PDF Methods Section:

Raw X-ray diffraction data for each sample were corrected for the effects of background, multiple scattering, container scattering, Compton scattering, fluorescence and absorption using the GudrunX programme[1,2]. The resulting total scattered intensity per atom, $\frac{1}{N}\mathrm{d}\sigma/\mathrm{d}\Omega$, is converted[3] to the distinct total scattering structure factor, $S(Q)$, due to atom-atom correlations, which will oscillate about a baseline determined by the coherent self-scattering. A 'sharpening' term is also introduced to enhance scattering at high Q:

$$S(Q) = \frac{\left[\frac{1}{N}\frac{\mathrm{d}\sigma}{\mathrm{d}\Omega} - \sum_{i=1}^{n} c_i f_i(Q)^2\right]}{\sum_{i=1}^{n} c_i f_i(Q)^2} \qquad (1)$$

Where there are N atoms in the sample, c_i is the proportion of element i and $f_i(Q)$ is the X-ray atomic form factor of element i [4]. Q is the scattering vector determined by the X-ray wavelength, λ, and the scattering angle 2θ:

$$Q = \frac{4\pi \sin\theta}{\lambda} \qquad (2)$$

For a non-interacting mixture of M phases the total intensity is assumed to be the weighted sum of intensities of each phase (not accounting for attenuation and the presence of interfaces)[5];

$$\frac{\mathrm{d}\sigma}{\mathrm{d}\Omega} = \sum_{k=1}^{M} \frac{\mathrm{d}\sigma}{\mathrm{d}\Omega}^{k} \qquad (3)$$

Where $\frac{\mathrm{d}\sigma}{\mathrm{d}\Omega}^{k}$ is the total scattered intensity of the kth phase in the multiphase sample, which is obtained from a measurement of $\frac{1}{N}\mathrm{d}\sigma/\mathrm{d}\Omega$ from a sample of phase k on its own, i.e. $\frac{\mathrm{d}\sigma}{\mathrm{d}\Omega}^{k} = N_k\left(\frac{1}{N}\mathrm{d}\sigma/\mathrm{d}\Omega\right)_k$ where N_k is the number of atoms of phase k within the multiphase sample. However due to the difficulty of placing X-ray scattering data on an absolute scale[2] a set of additional scaling factors are introduced:

$$a_k I_k^{\mathrm{Norm}} = \frac{\mathrm{d}\sigma}{\mathrm{d}\Omega}^{k} \qquad (4)$$

Where I_k^{Norm} is the approximately normalised intensity and a_k is a constant close to unity. Equation (3) now becomes;

$$a_{\text{Expt.}} I_{\text{Expt.}}^{\text{Norm}} = \sum_{k=1}^{M} a_k I_k^{\text{Norm}} \tag{5}$$

In order to produce the expected scattering intensity of a non-interacting mixture the experimental total scattering intensities per atom of the pure a_gZIF-62 and inorganic glasses are recovered from their respective S(Q) via equation (1) and added together weighted by their atomic proportions according to (5). The total scattered intensity for the composite samples, (a_gZIF-62)$_{0.5}$(Inorganic Glass)$_{0.5}$ – 1 min and (a_gZIF-62)$_{0.5}$(Inorganic Glass)$_{0.5}$ – 30 min, the experimental total scattering, are also recovered from their respective $S(Q)$s via (1).

The difference between measured intensity from a sample and the calculated intensity from a non-interacting mixture of the same chemistry therefore represents changes in the diffracted intensity resulting from interaction of the a_gZIF-62 and inorganic glass phases in the composite. This difference is then re-sharpened by dividing through by $\sum_{i=1}^{n} c_i f_i(Q)^2$:

$$S(Q)^{\text{Diff}} = \frac{a_{\text{Expt.}} I_{\text{Expt.}}^{\text{Norm}} - \left(a_{\text{Inorg.}} I_{\text{Inorg.}}^{\text{Norm}} + a_{\text{ZIF}} I_{\text{ZIF}}^{\text{Norm}}\right)}{\sum_{i=1}^{n} c_i f_i(Q)^2} \tag{6}$$

In this paper the values of a_k where determined such that the difference between the calculated mixture and the experimentally measured scattering, weighted to produce smaller differences at higher Q-values, was minimised for each different inorganic glass composition. One $a_{Expt.}$ scale factor was kept fixed at unity as a reference point.

$S(Q)^{Diff}$ is then Fourier transformed in the usual way to produce $G(r)^{Diff}$ [6]. Where the $G(r)^{Diff}$ now represents a weighted histogram of the distribution of atom-atom distances in the composite sample which are due to interactions between the inorganic glass and a_gZIF-62, such that peaks represent new correlations formed as a result of the interaction between the two phases; either new bonds formed at the interface or changes in the structure of either phase due to the presence of the other. Finally in order to aid visualisation of new correlations across the full range of real space an alternative form of the usual $D(r)$ function is plotted:

$$D(r)^{\text{Diff}} = \left(G(r)^{\text{Diff}} - 1\right)r \tag{7}$$

Due to the difficulty of putting X-ray total scattering data on an absolute scale $S(Q)^{\text{Diff}}$ can contain residual scattering from the individual phases. The size and shape of this 'erroneous' difference is dependent on the scale factors (a_k) used in equation (4). The presence of these additional features in the $S(Q)^{\text{Diff}}$ complicates interpretation of the $G(r)^{\text{Diff}}$ and $D(r)^{\text{Diff}}$; underweighting of the total scattering from a constituent phase will lead to positive peaks from that phase in the $G(r)^{\text{Diff}}$ and $D(r)^{\text{Diff}}$ as residual scattering from that phase remains in the $S(Q)^{\text{Diff}}$ and conversely overweighting will lead to negative peaks in the $G(r)^{\text{Diff}}$ and $D(r)^{\text{Diff}}$ as the total scattering from a phase is over-removed from the $S(Q)^{\text{Diff}}$.

As a result of this interpretation of the real space data should always be done in the context of the correlations expected from the phases in the calculated mixture.

Supplementary Figures

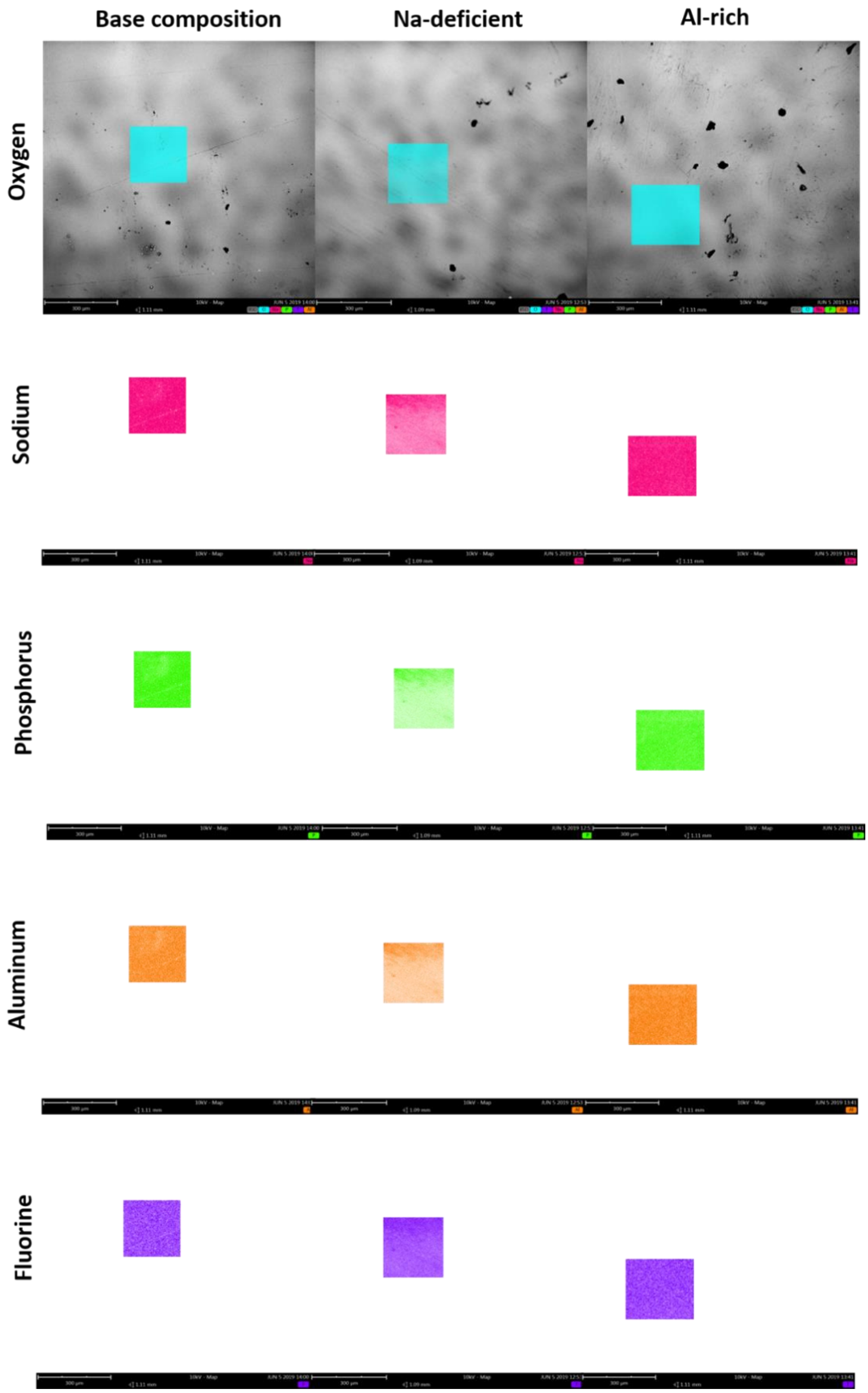

Supplementary Fig. 1. Individual elemental maps from EDX on inorganic glasses: base composition, Na-deficient and Al-rich.

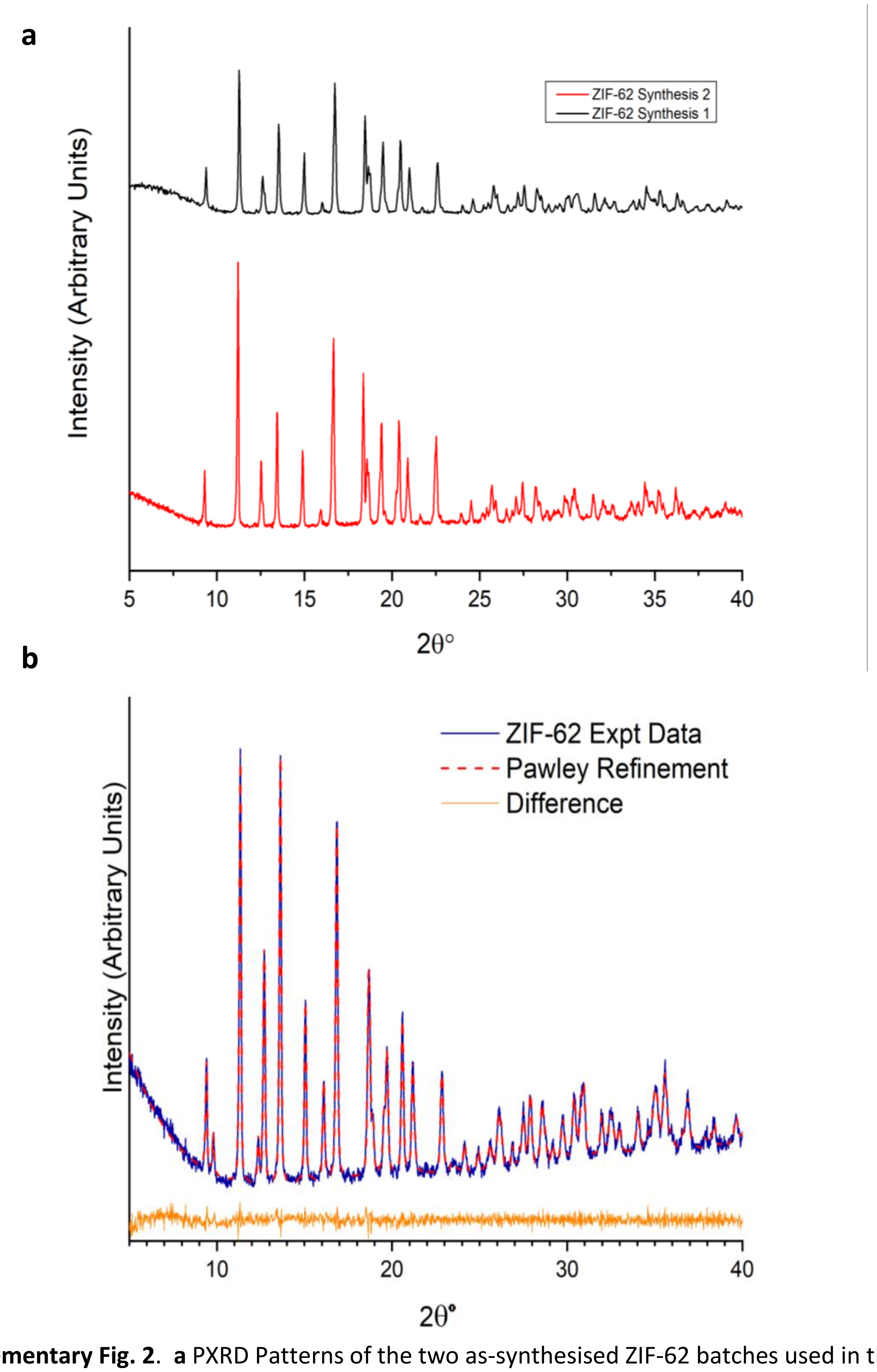

Supplementary Fig. 2. **a** PXRD Patterns of the two as-synthesised ZIF-62 batches used in this study. The ZIF-62 synthesis 1 pattern was collected on a B1 (BB) Bruker D8 DAVINCI diffractometer using Cu Kα (λ = 1.5418 Å) radiation and a LynxEye position sensitive detector in Bragg–Brentano parafocussing geometry. A 5-70 ˚ 2θ angular range was used with a step size of 0.04. An offset has been added to improve readability. **b** Pawley refinement of the evacuated ball milled ZIF-62 sample. R_{wp} = 5.535. Refined unit cell values: a = 15.486 ± 0.005 Å, b = 15.545 ± 0.005 Å, c = 17.984 ± 0.004 Å. Initial parameters were obtained from Ref[7].

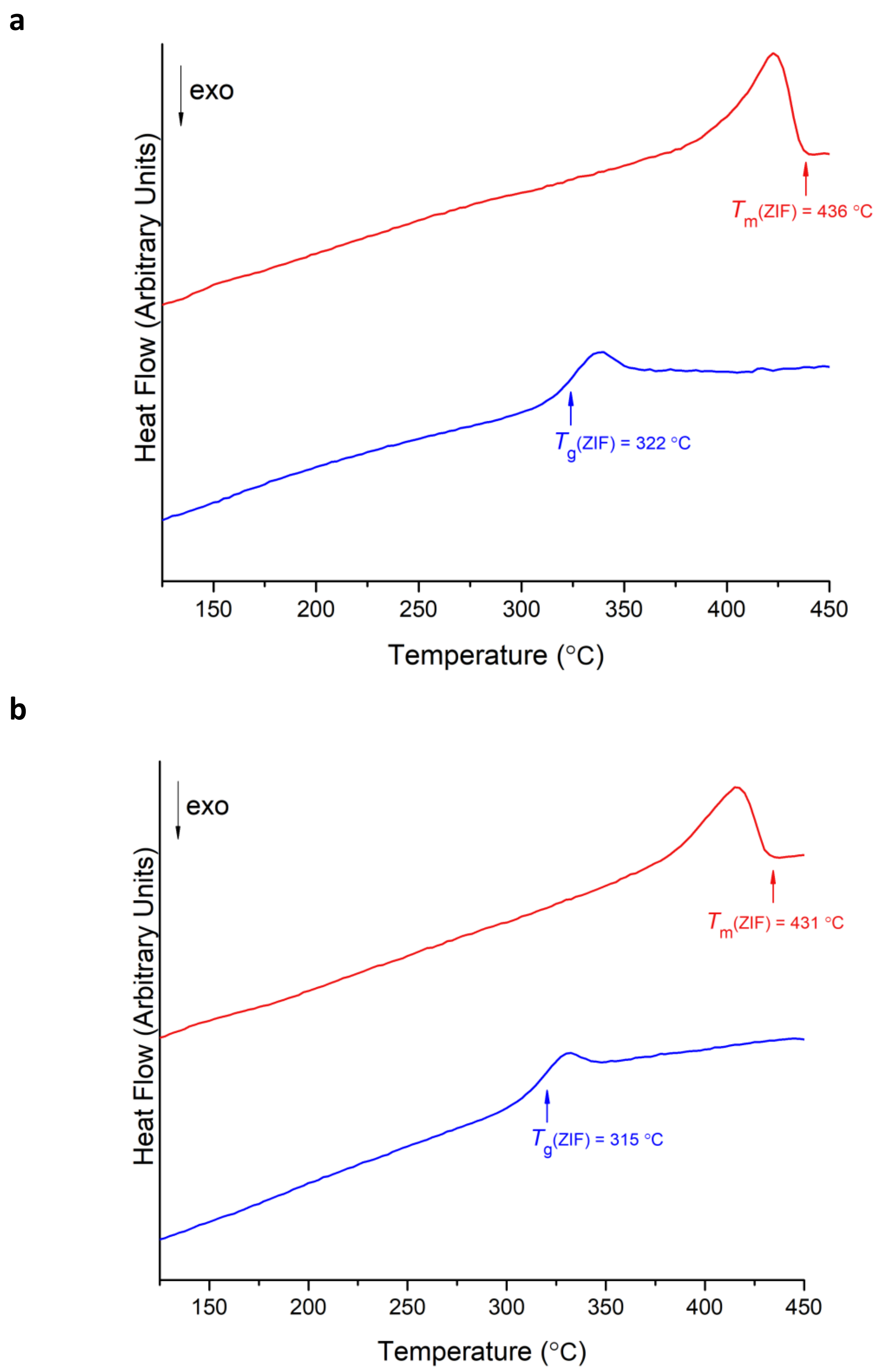

Supplementary Fig. 3. Thermal response of ZIF-62 control on the first (red) and second (blue) DSC heating scans. **a** after 1 minute. **b** after 30 minutes (bottom) at 450˚C. An offset has been added to improve readability.

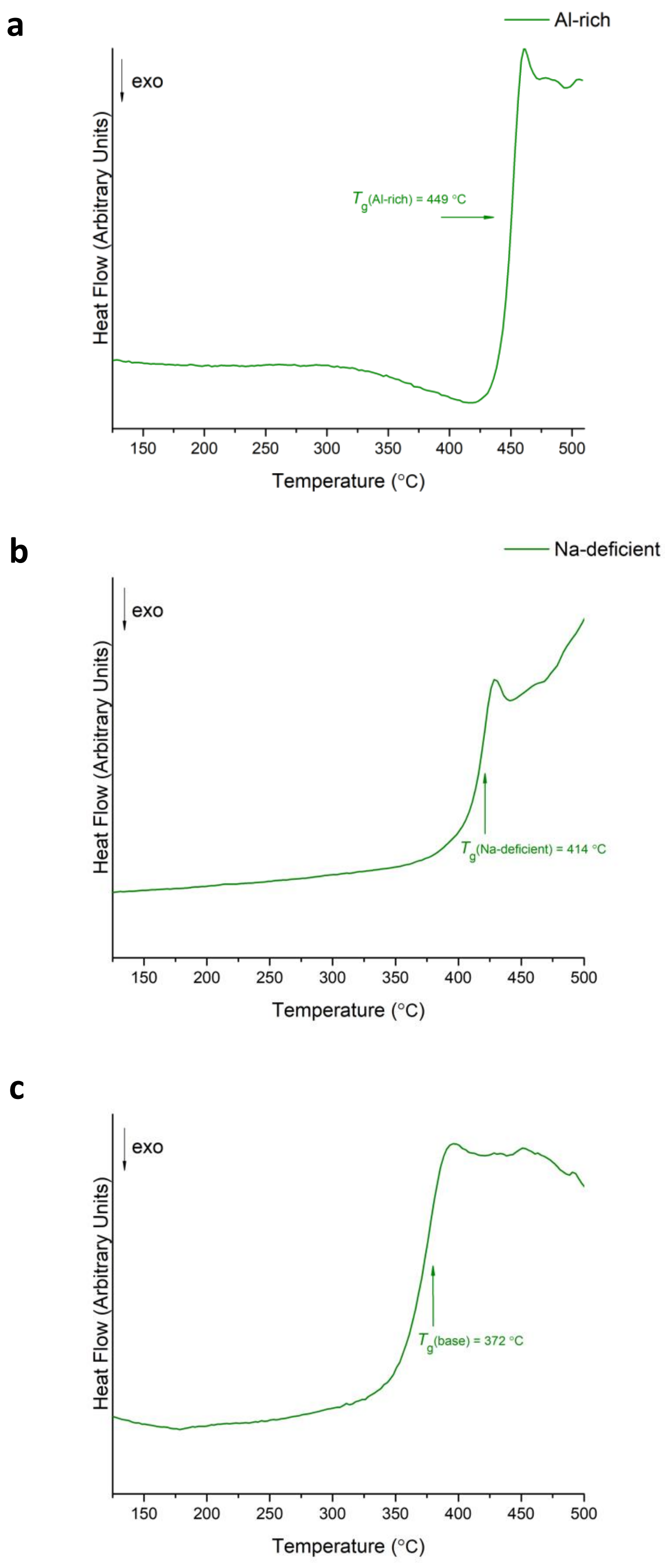

Supplementary Fig. 4. Thermal response of the inorganic controls. **a** Al-rich. **b** Na-deficient. **c** Base.

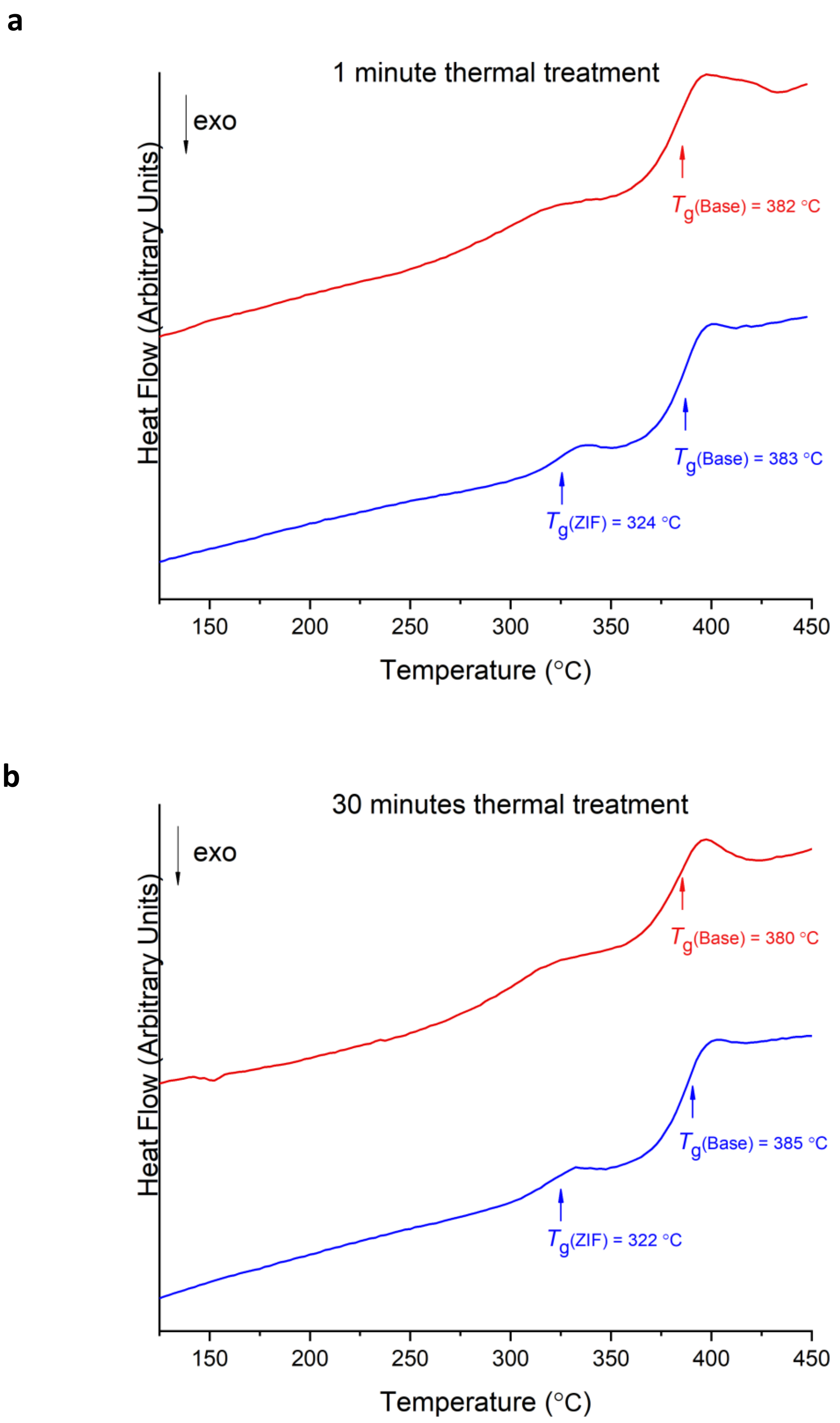

Supplementary Fig. 5. Thermal response of (ZIF-62)(base)(50/50) on the first (red) and second (blue) DSC heating scans. **a** after 1 minute. **b** after bottom 30 minutes at 450˚C. An offset has been added to improve readability.

a

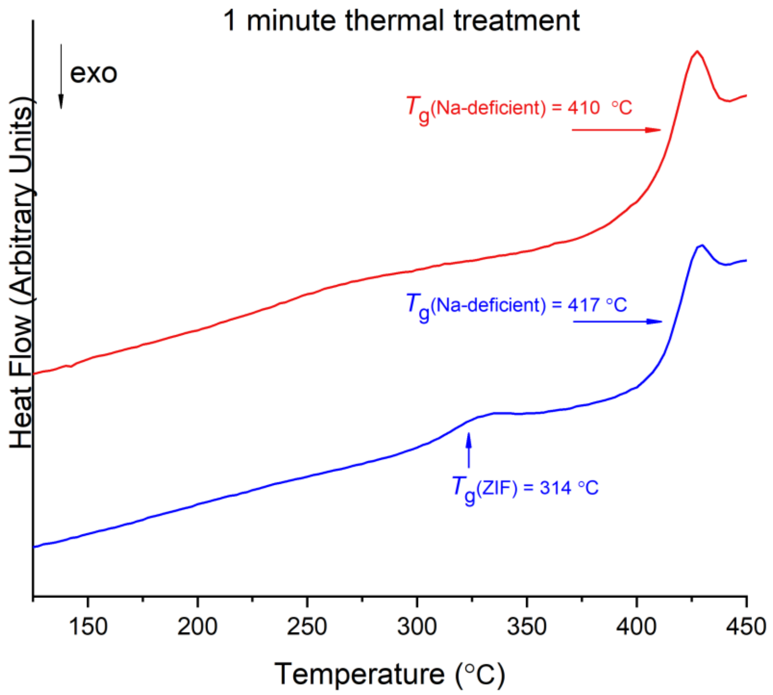

b

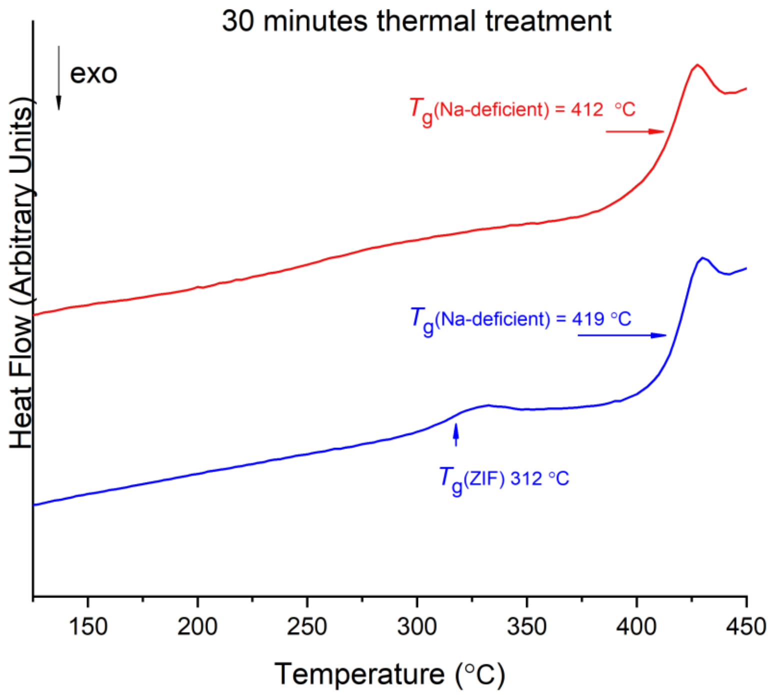

Supplementary Fig. 6. Thermal response of (ZIF-62)(Na-deficient)(50/50) on the first (red) and second (blue) DSC heating scans. **a** after 1 minute. **b** after 30 minutes at 450˚C. An offset has been added to improve readability.

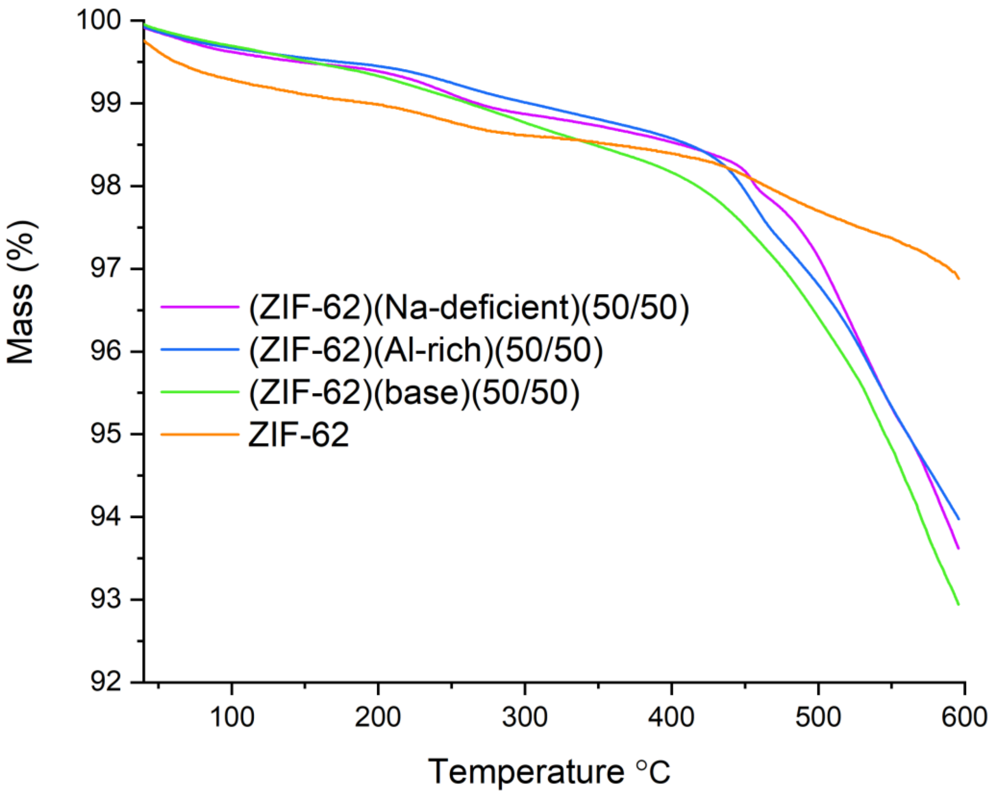

Supplementary Fig. 7. Thermogravimetric trace of the (ZIF-62)(Inorganic Glass)(50/50). All samples display less than 3 % mass loss below 450 ˚C.

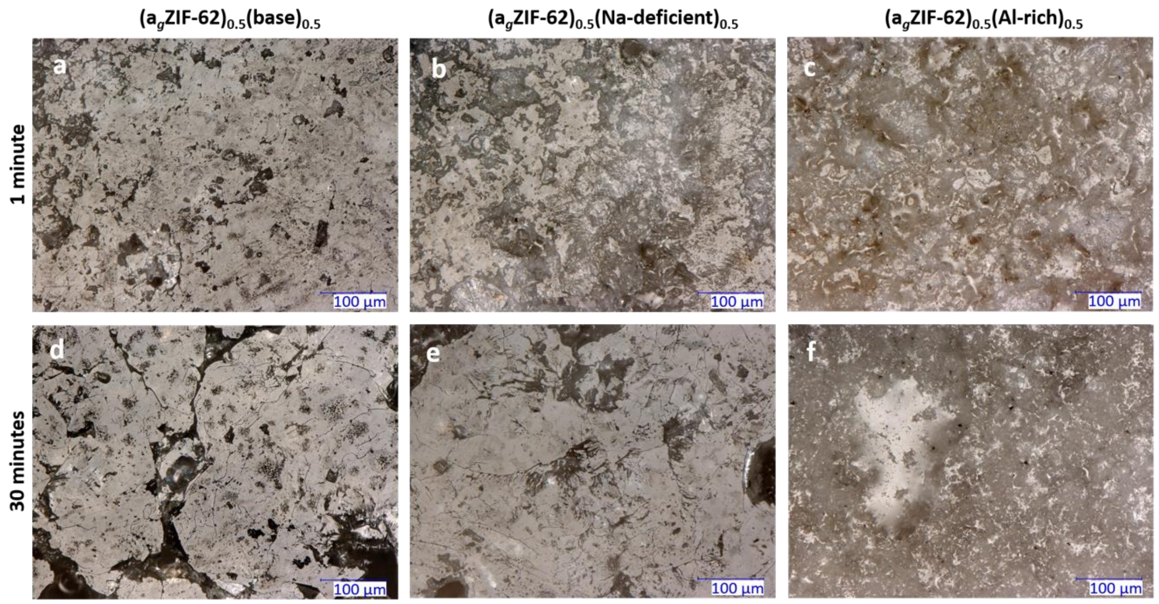

Supplementary Fig. 8. Top-illuminated confocal microscopy images of the (a$_g$ZIF-62)$_{0.5}$(Inorganic Glass)$_{0.5}$ compositions heat treated for 1 minute (top row) and 30 minutes (bottom row).

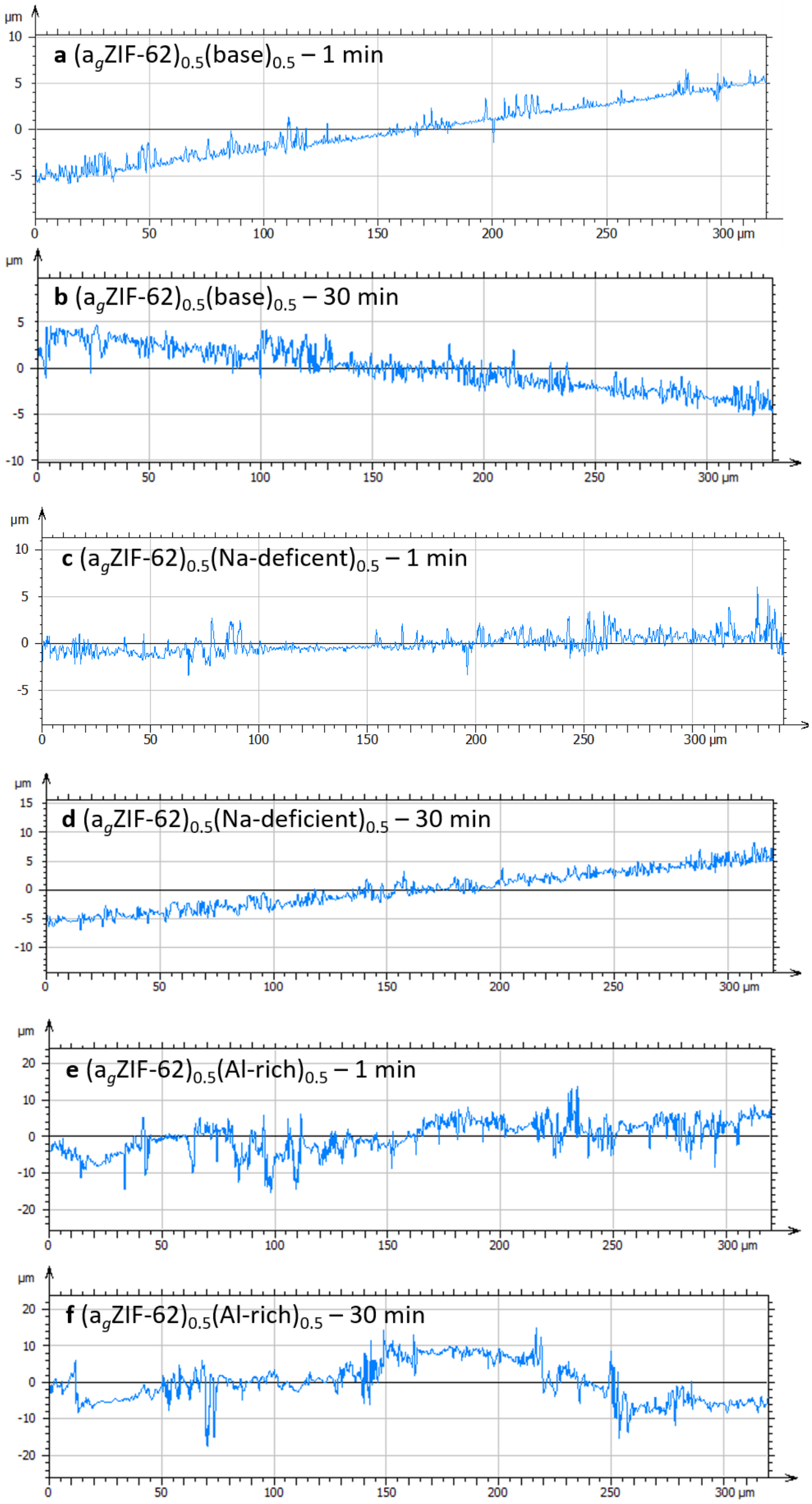

Supplementary Fig. 9. Roughness profiles of (a$_g$ZIF-62)$_{0.5}$(base)$_{0.5}$ – 1 min (a) and – 30 min (b) compared with (a$_g$ZIF-62)$_{0.5}$(Na-deficient)$_{0.5}$ – 1 min (c) and – 30 min (d) and (a$_g$ZIF-62)$_{0.5}$(Al-rich)$_{0.5}$ – 1 min (e) and – 30 min (f).

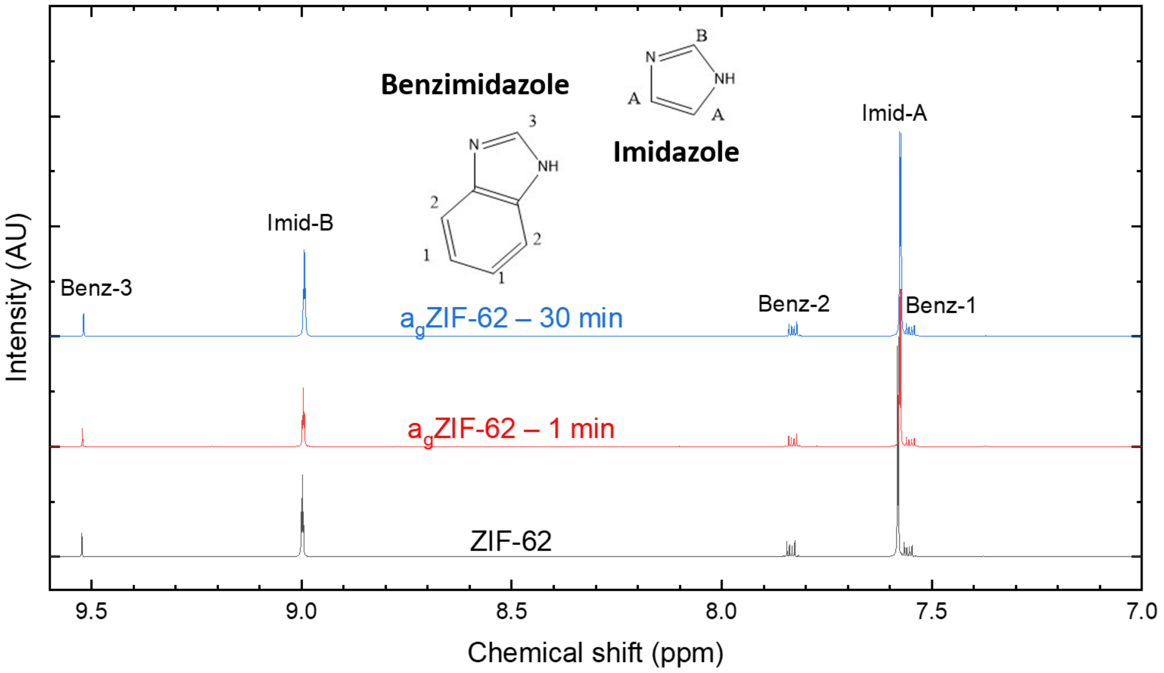

Supplementary Fig. 10. Liquid ^{1}H NMR spectra of crystalline and amorphous pure ZIF-62 controls (1 and 30 min heat treatments) with the labelled NMR peaks of the ZIF linkers inset. In ZIF-62, the anions imidazolate and benzimidazolate are present without the H. An offset has been added to improve readability.

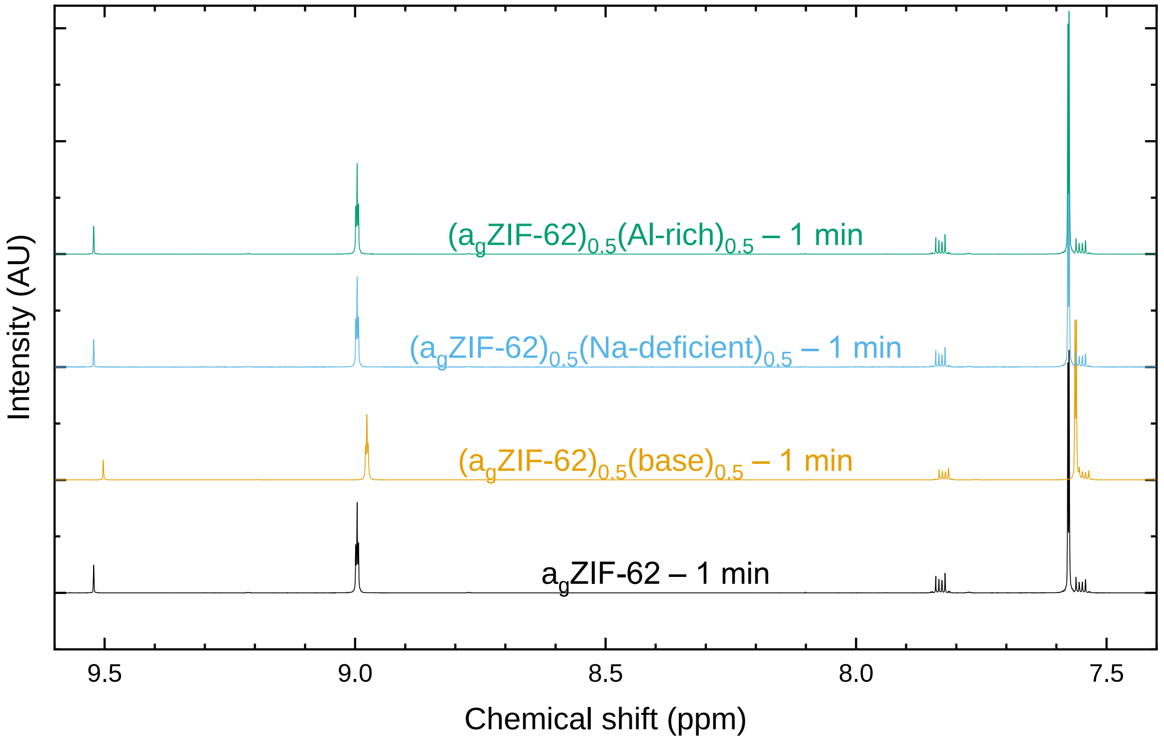

Supplementary Fig. 11. Liquid ^{1}H NMR spectra of (*ag*ZIF-62)(Inorganic Glass) composites treated for 1 minute. An offset has been added to improve readability.

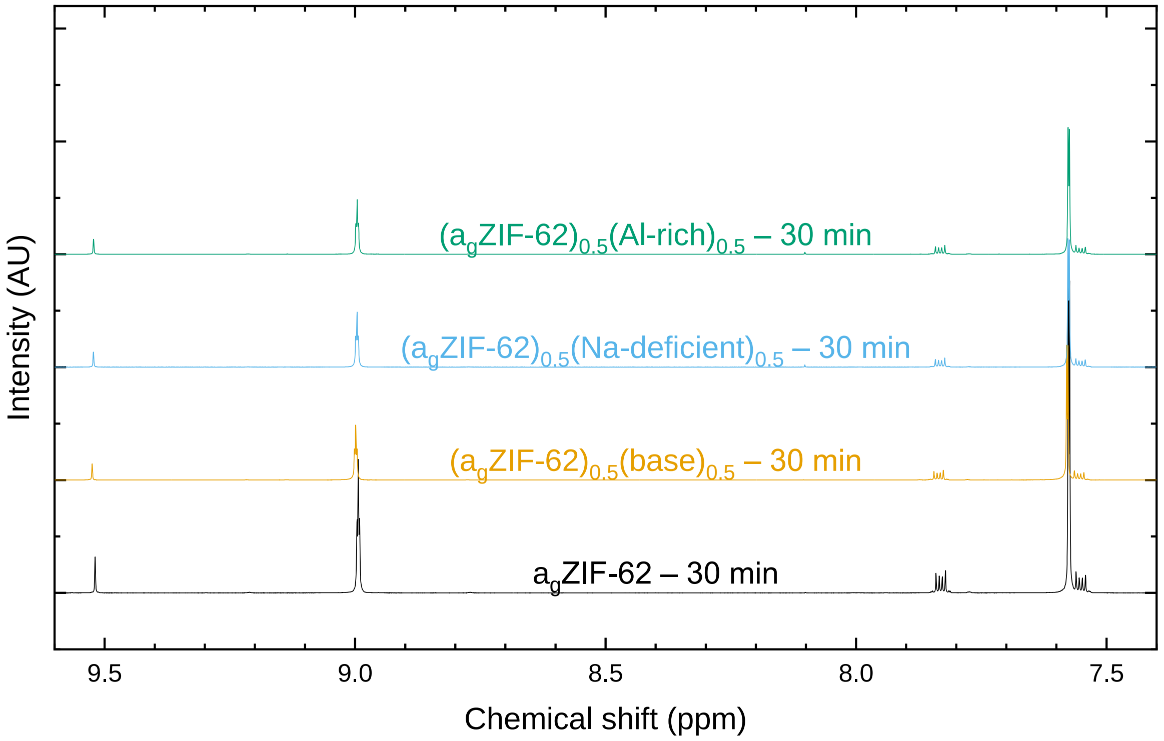

Supplementary Fig. 12. Liquid ^{1}H NMR spectra of (a_gZIF-62)(Inorganic Glass) composites treated for 30 minutes. An offset has been added to improve readability.

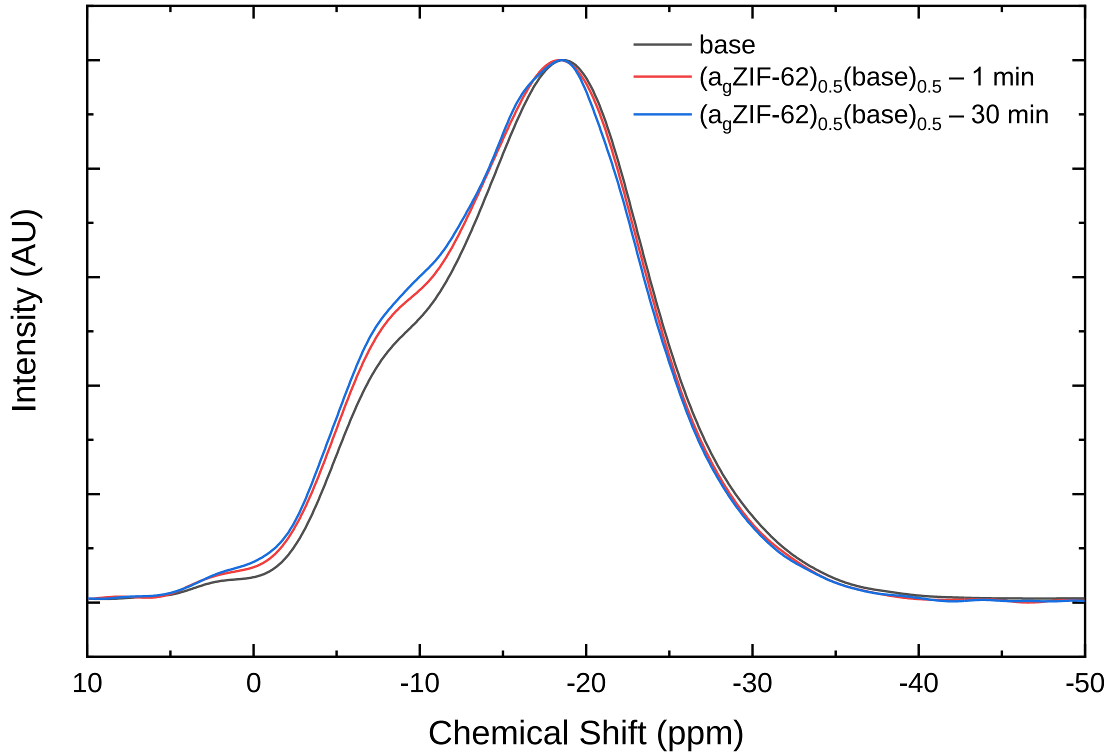

Supplementary Fig. 13. ^{31}P SS NMR of the inorganic glass (base) and the composites (a_gZIF-62)$_{0.5}$(base)$_{0.5}$ heat treated for 1 min and 30 min.

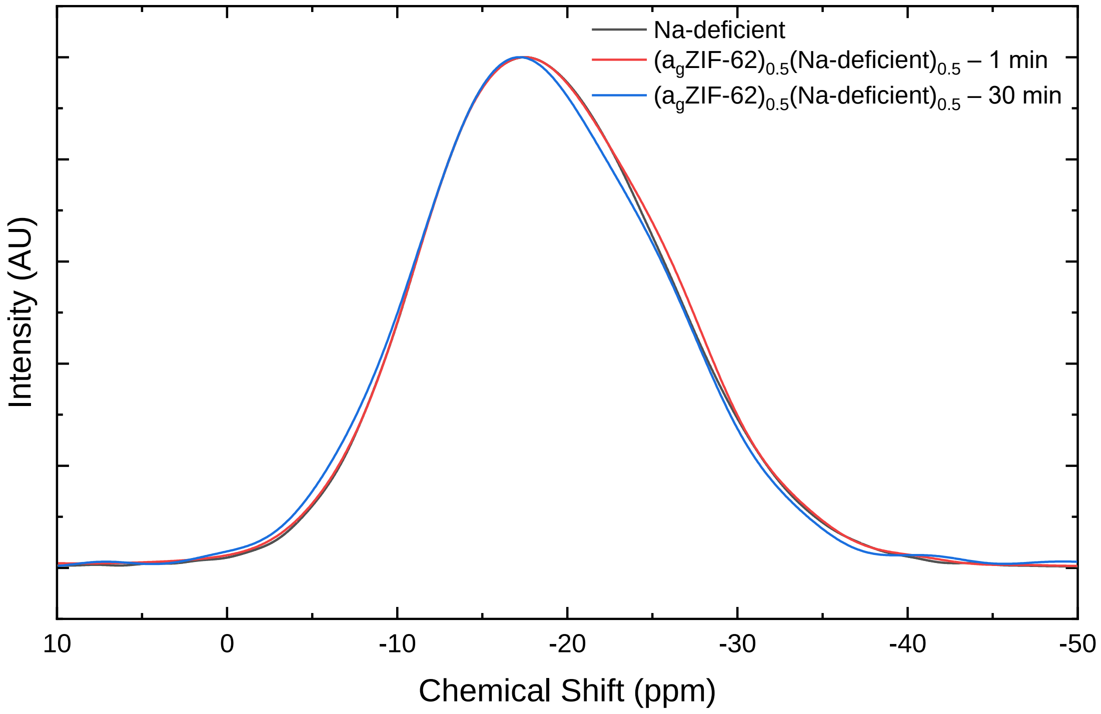

Supplementary Fig. 14. ^{31}P SS NMR of the inorganic glass (Na-deficient) and the composites (a_gZIF-62)$_{0.5}$(Na-deficient)$_{0.5}$ heat treated for 1 min and 30 min.

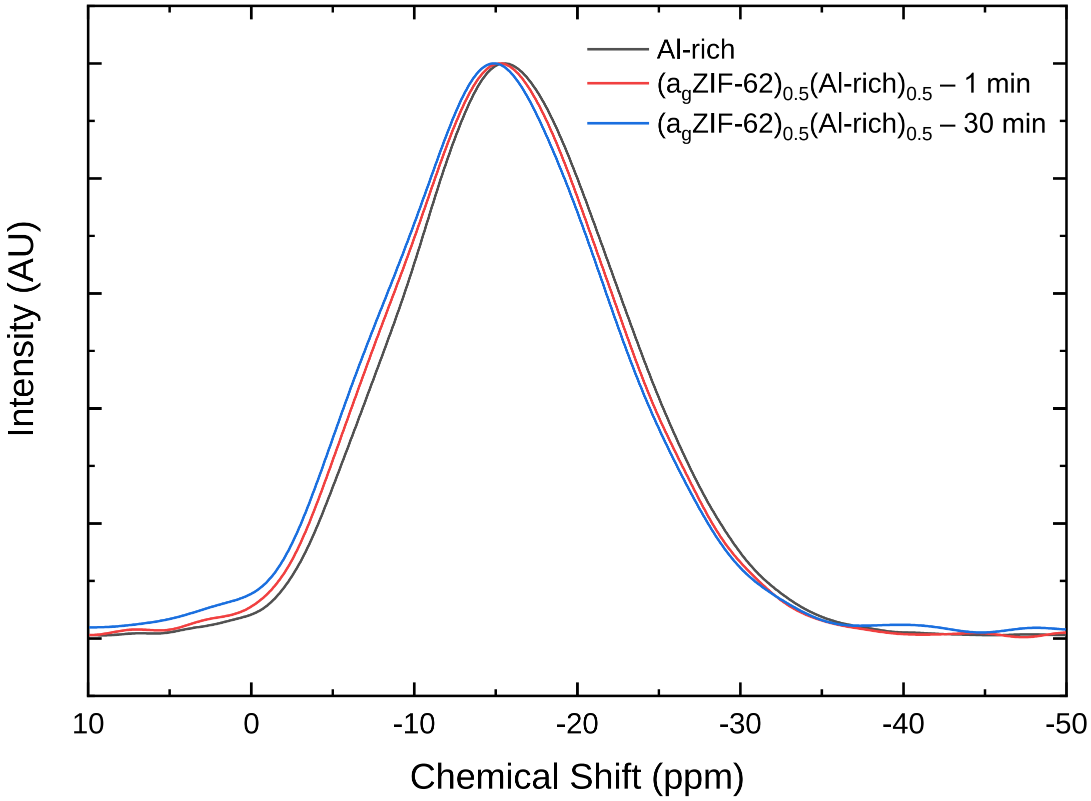

Supplementary Fig. 15. ^{31}P SS NMR of the inorganic glass (Al-rich) and the composites (a_gZIF-62)$_{0.5}$(Al-rich)$_{0.5}$ heat treated for 1 min and 30 min.

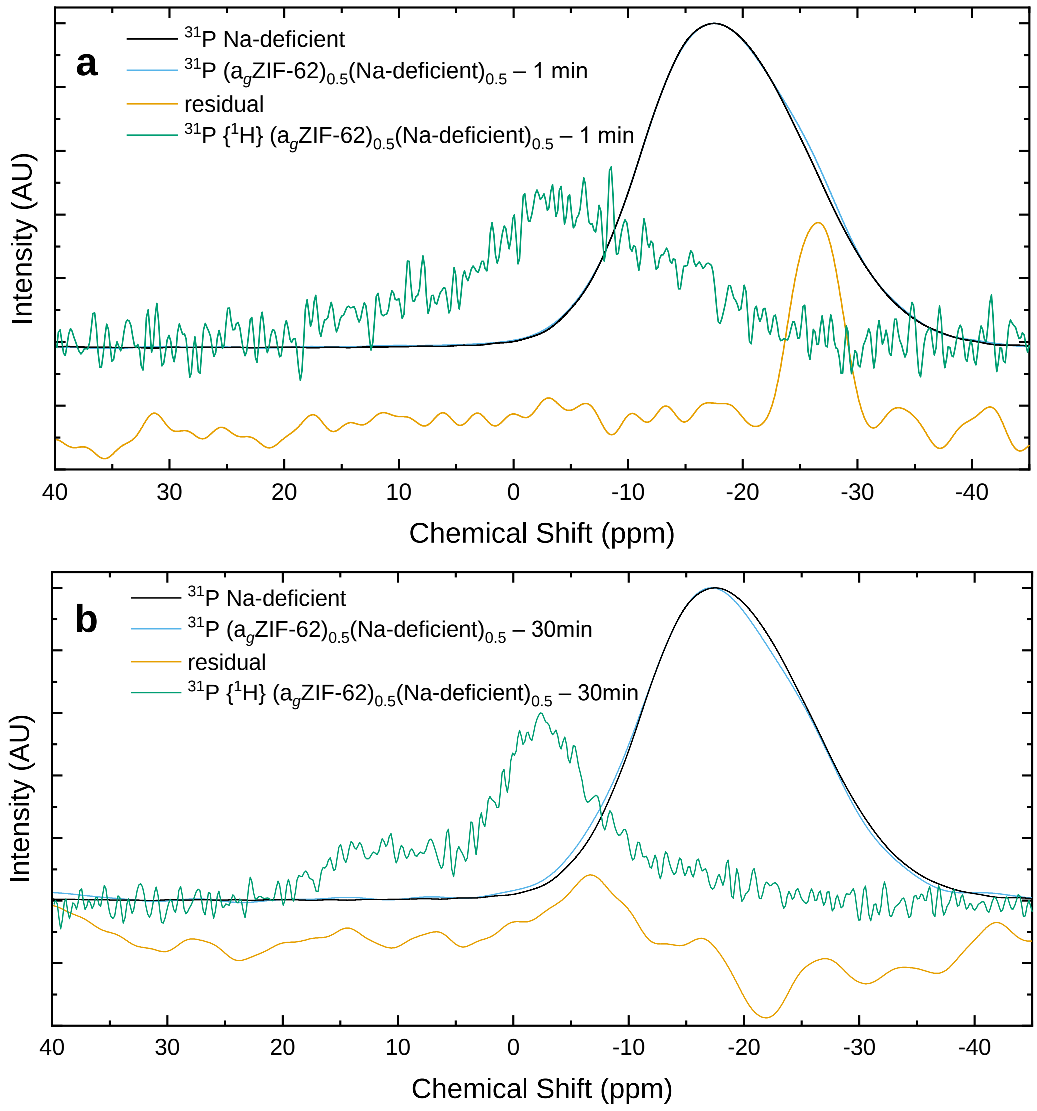

Supplementary Fig. 16. ^{31}P SS NMR and $^{31}P\{^{1}H\}$ CP NMR of the inorganic glass (Na-deficient) and the composites $(a_gZIF\text{-}62)_{0.5}(Na\text{-deficient})_{0.5}$ heat treated for 1 min (a) and 30 min (b).

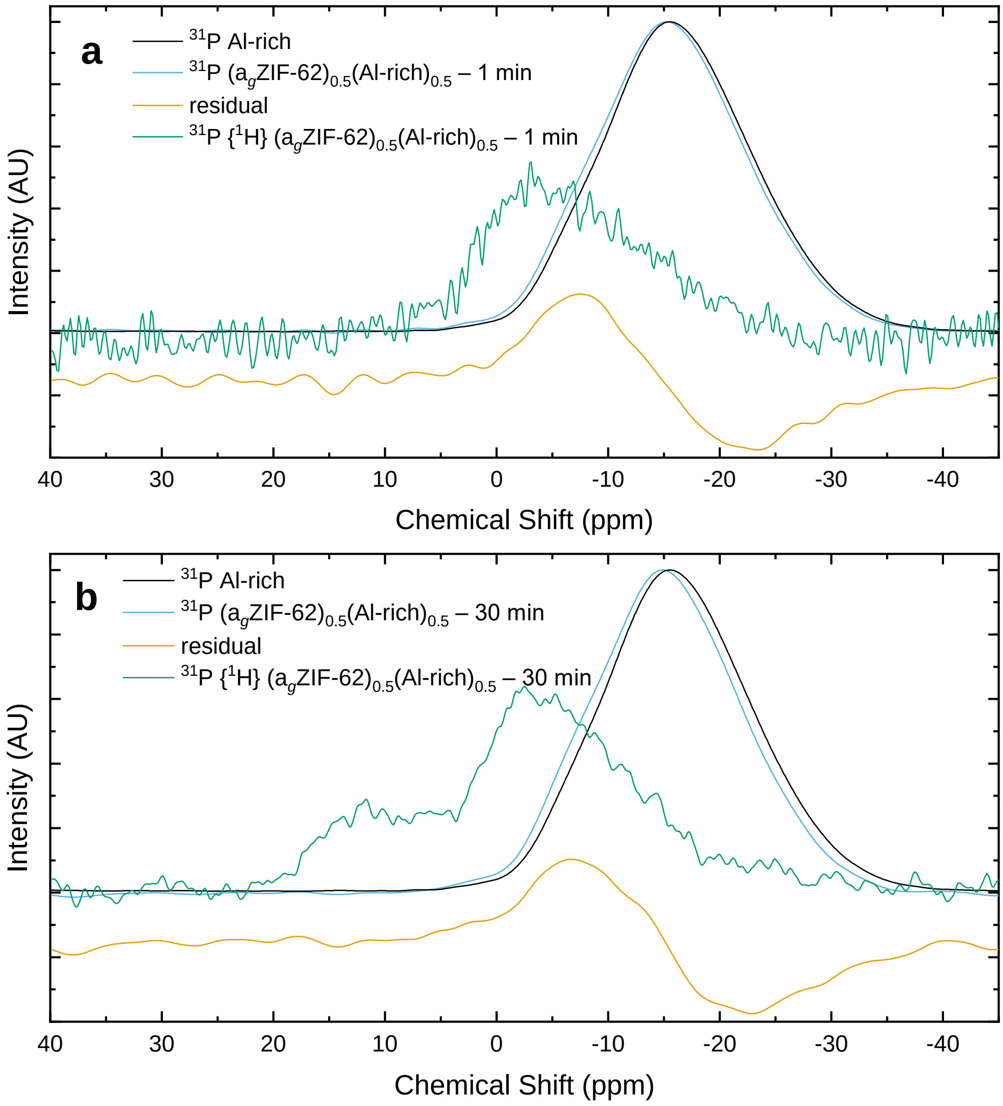

Supplementary Fig. 17. ^{31}P SS NMR and $^{31}P\{^{1}H\}$ CP NMR of the inorganic glass (Al-rich) and the composites $(a_gZIF\text{-}62)_{0.5}(Al\text{-}rich)_{0.5}$ heat treated for 1 min (a) and 30 min (b).

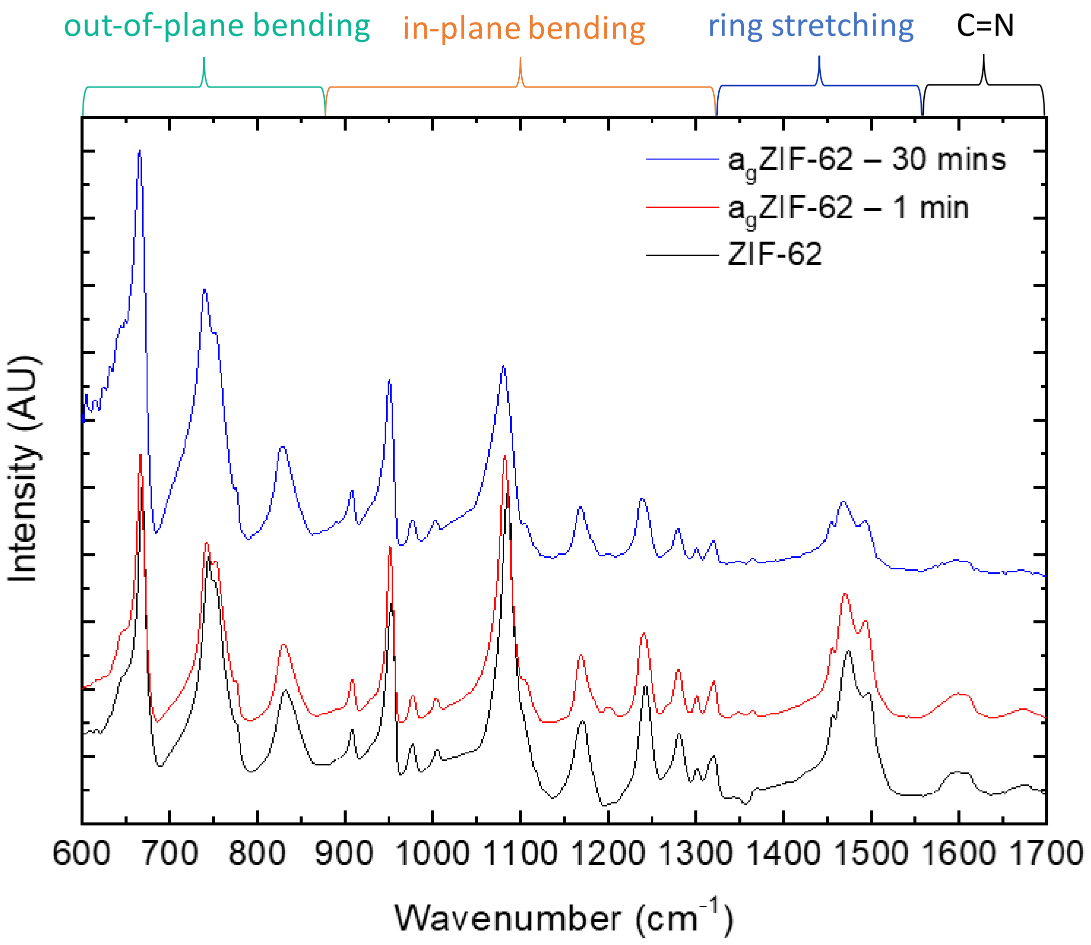

Supplementary Fig. 18. ATR-IR spectra of pure ZIF-62 controls, crystalline and heat treated for 1 and 30 minutes. An offset has been added to improve readability.

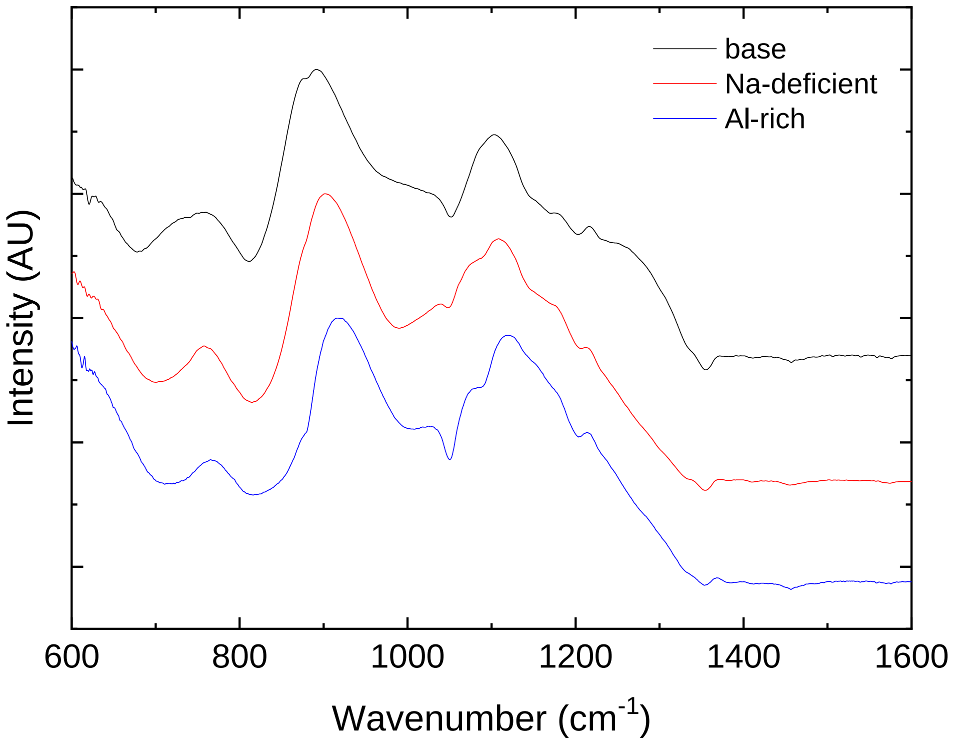

Supplementary Fig. 19. ATR-IR spectra of pure inorganic glasses used in composites. An offset has been added to improve readability.

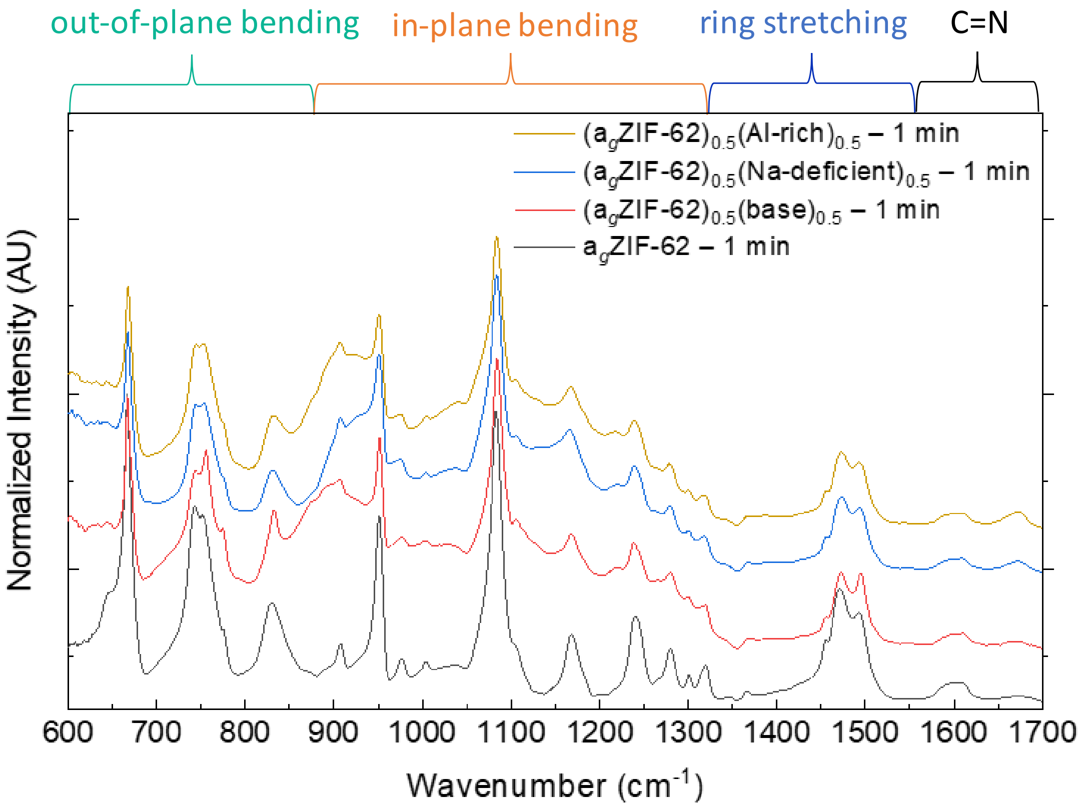

Supplementary Fig. 20. ATR-IR spectra of pure ZIF-62 control and composites heat treated for 1 min. An offset has been added to improve readability.

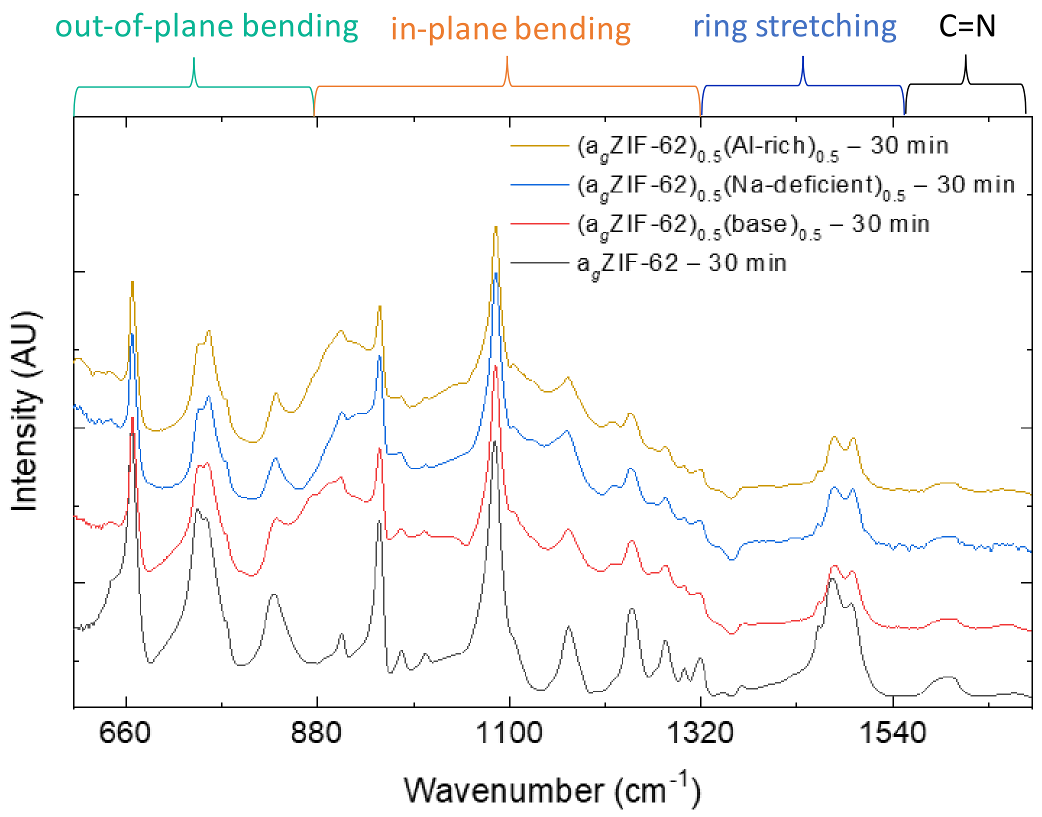

Supplementary Fig. 21. ATR-IR spectra of pure ZIF-62 control and composites heat treated for 30 min. An offset has been added to improve readability.

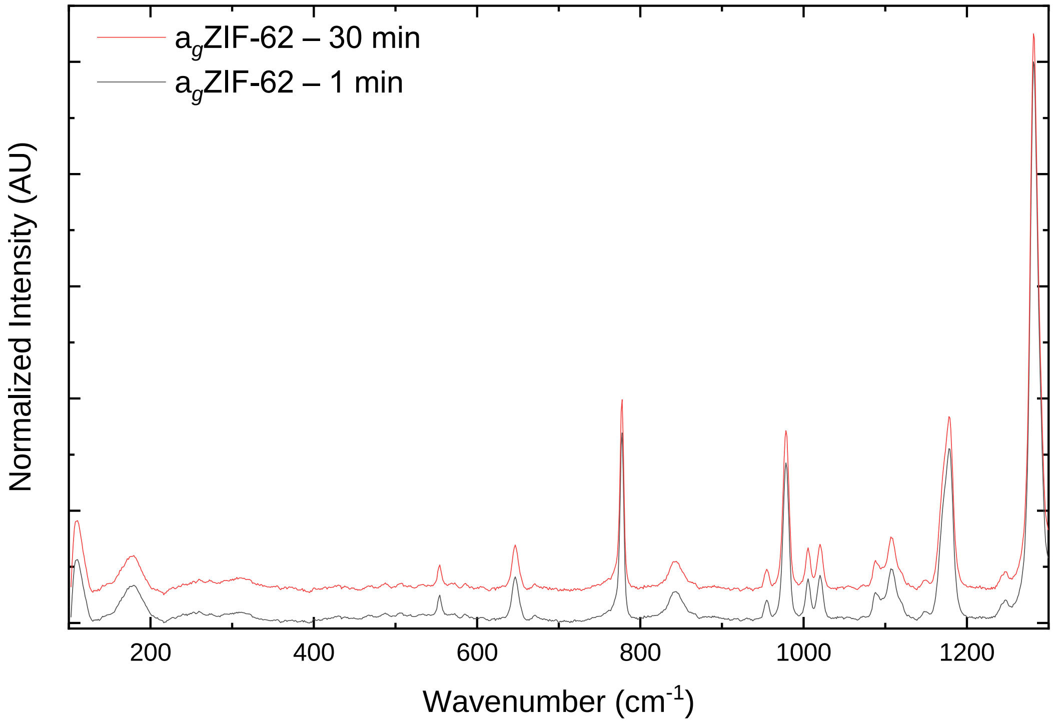

Supplementary Fig. 22. Raman spectra of the heat treated ZIF-62 control samples, a_gZIF-62 – 1 min and – 30 mins. An offset has been added to improve readability.

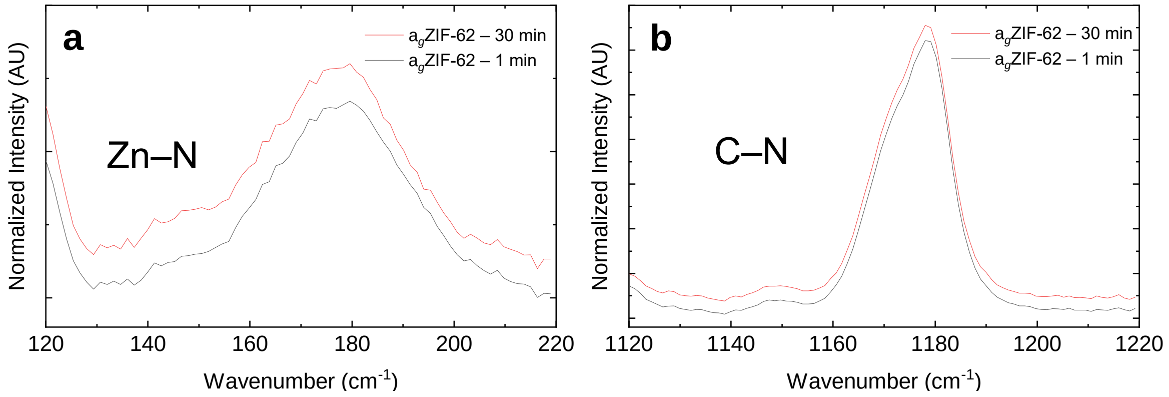

Supplementary Fig. 23. Magnified regions of the Raman spectra of the heat treated ZIF-62 control samples, **a** a_gZIF-62 – 1 min (a) and **b** – 30 mins (b). An offset has been added to improve readability.

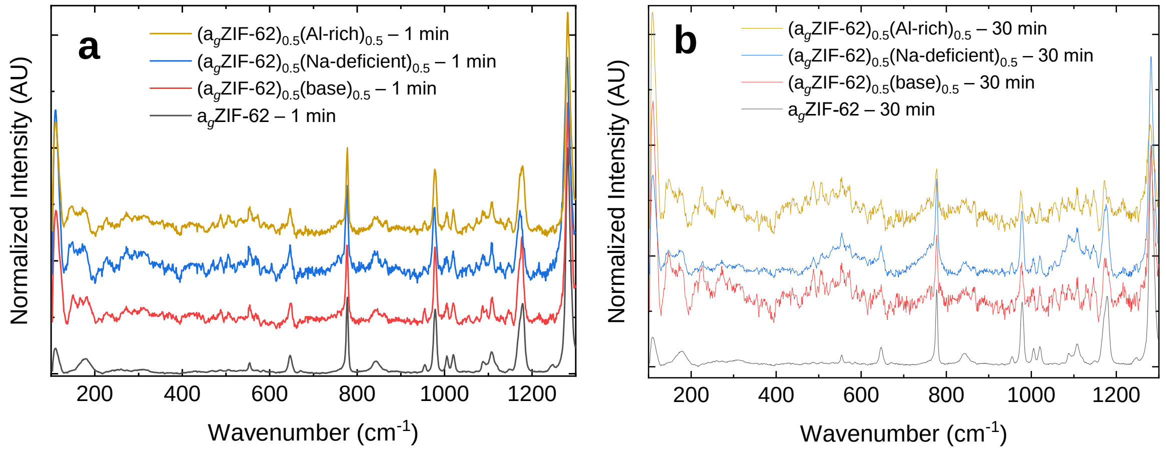

Supplementary Fig. 24. Raman spectra from the $(a_gZIF\text{-}62)_{0.5}(Inorganic\ Glass)_{0.5}$ – 1 min (a) and – 30 min samples (b). An offset has been added to improve readability.

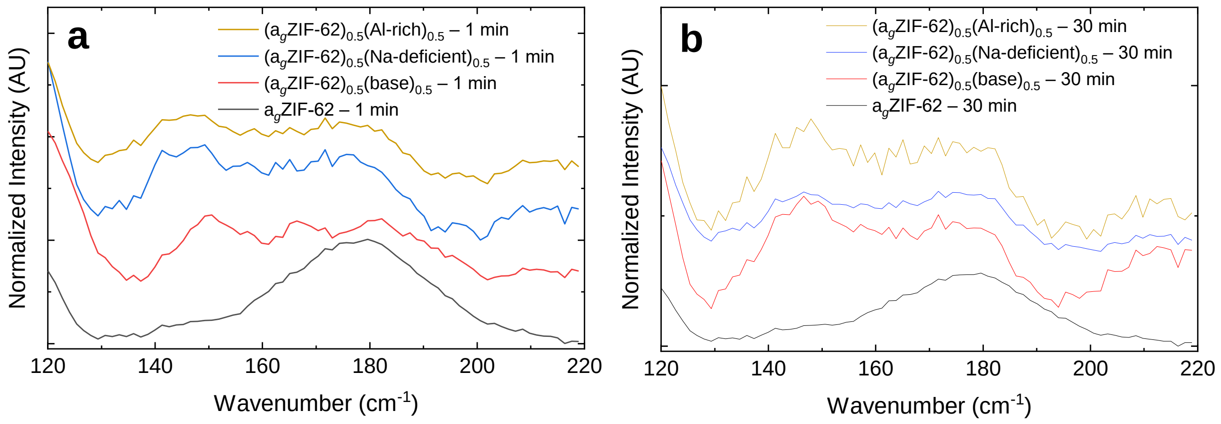

Supplementary Fig. 25. Micro-Raman spectra of Zn—N peak (ca. 175 cm^{-1}) peak of $(a_gZIF\text{-}62)_{0.5}(Inorganic\ Glass)_{0.5}$ – 1 min (a) and – 30 min samples (b). An offset has been added to improve readability.

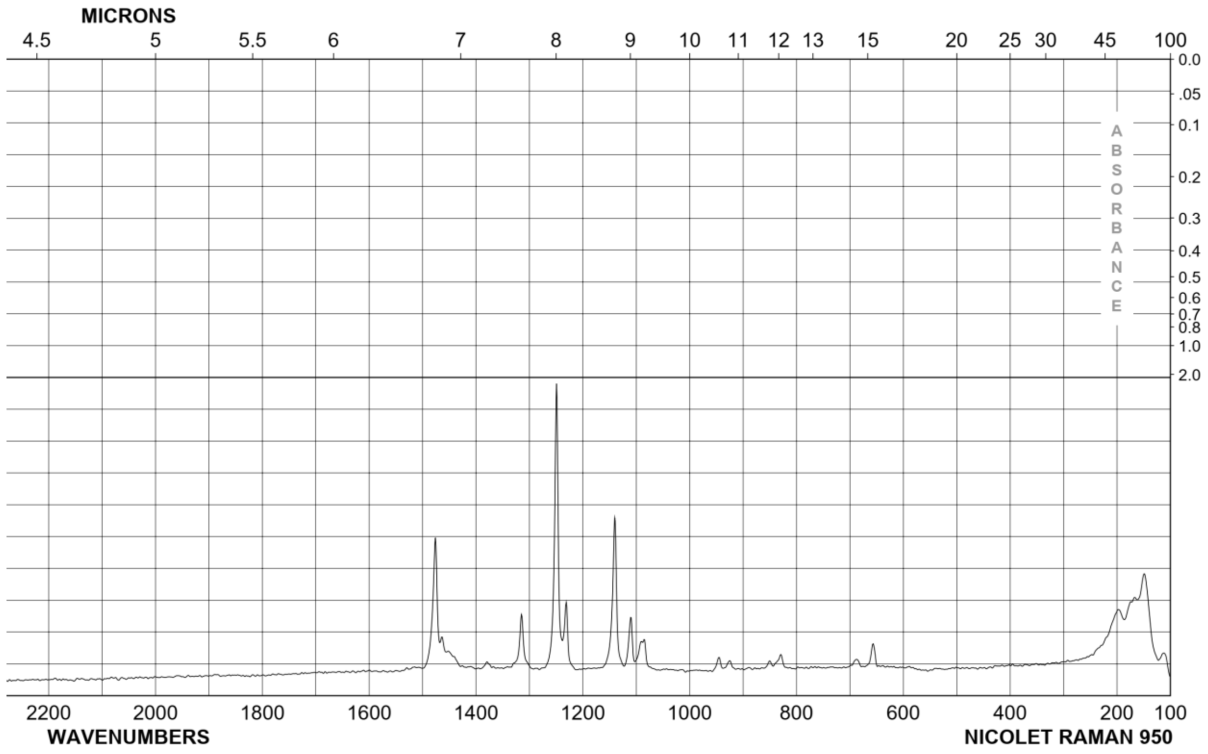

Supplementary Fig. 26: Raman spectra of sodium imidazole derivative from Sigma-Aldrich.[8]

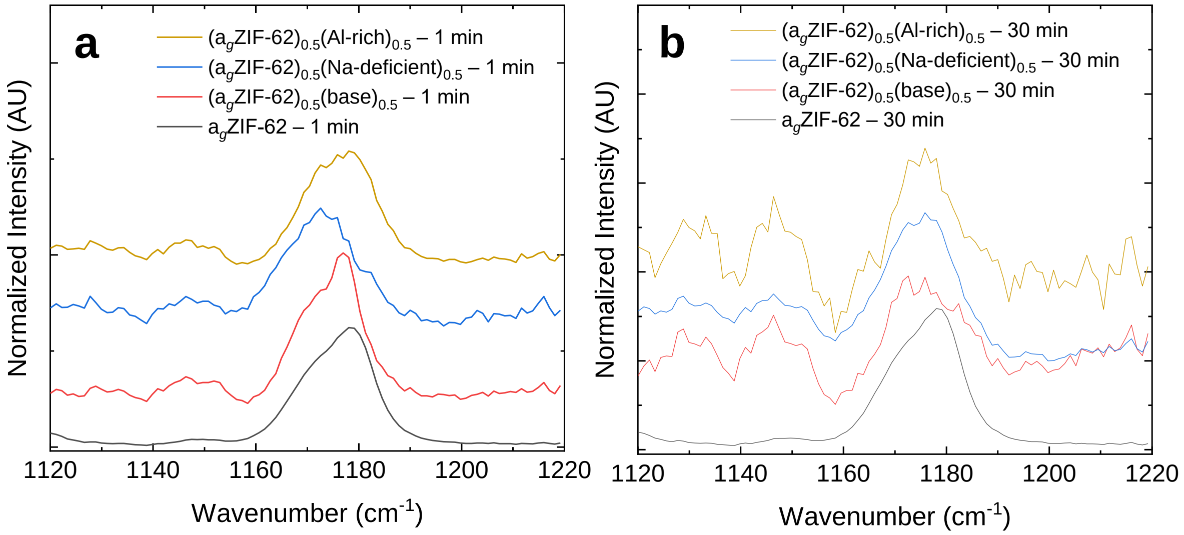

Supplementary Fig. 27. Micro-Raman spectra of C—N peak (ca. 1175 cm-1) peak of (a_gZIF-62)$_{0.5}$(Inorganic Glass)$_{0.5}$ – 1 min (a) and – 30 min samples (b). An offset has been added to improve readability.

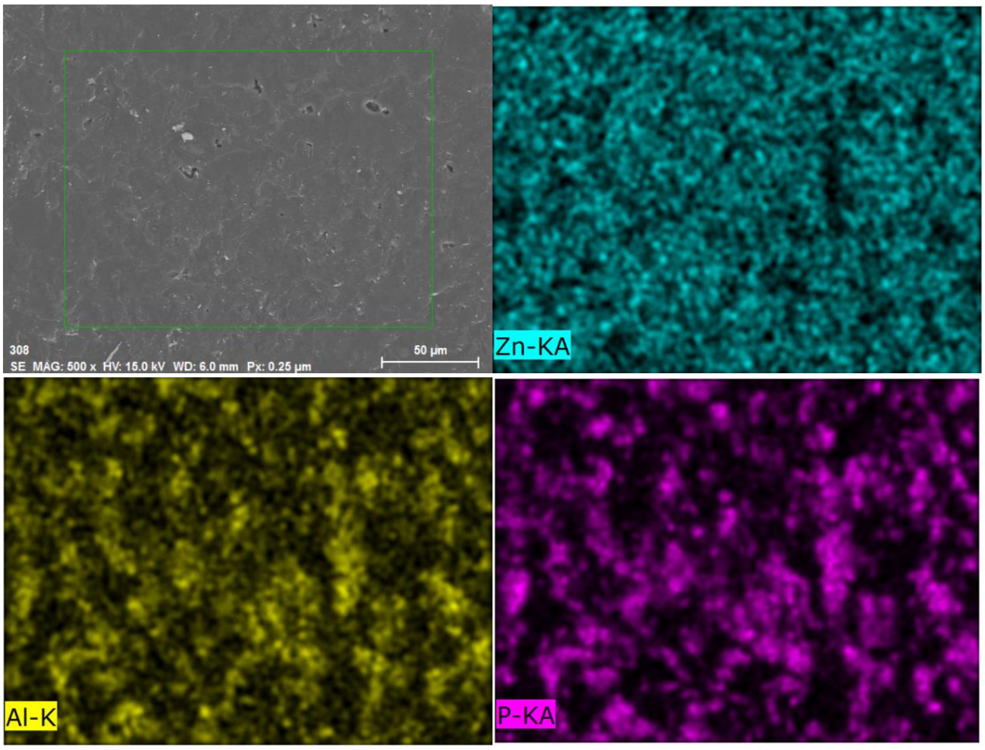

Supplementary Fig. 28. EDS of $(a_gZIF\text{-}62)_{0.5}(base)_{0.5}$ – 1 min.

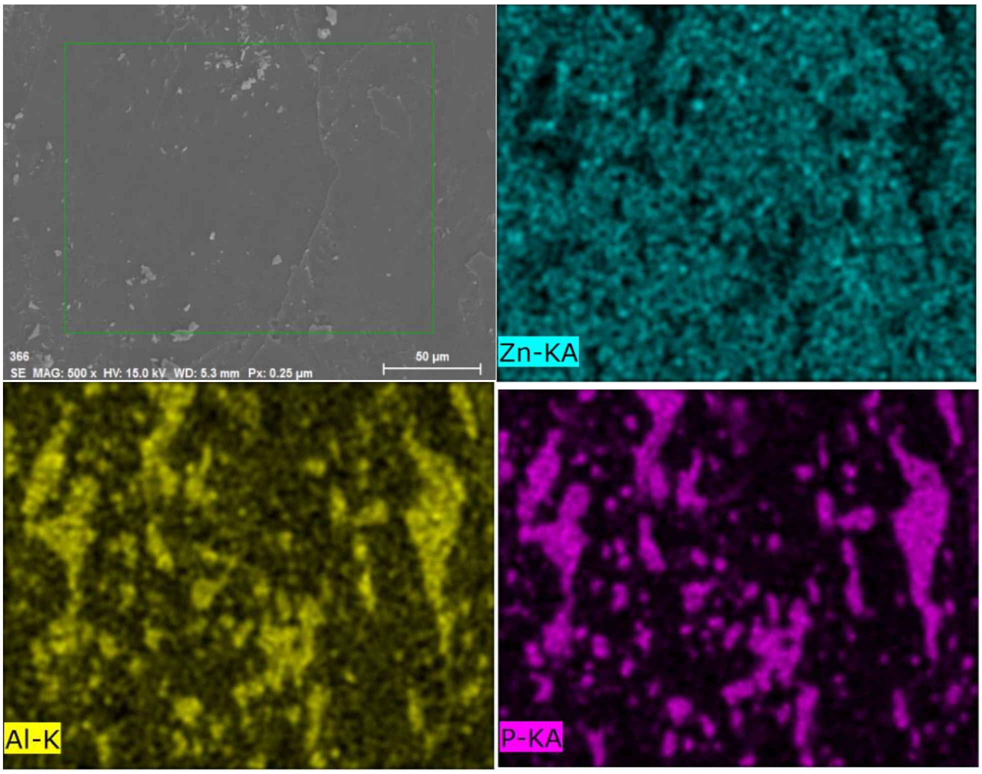

Supplementary Fig. 29. EDS of $(a_gZIF\text{-}62)_{0.5}(base)_{0.5}$ – 30min.

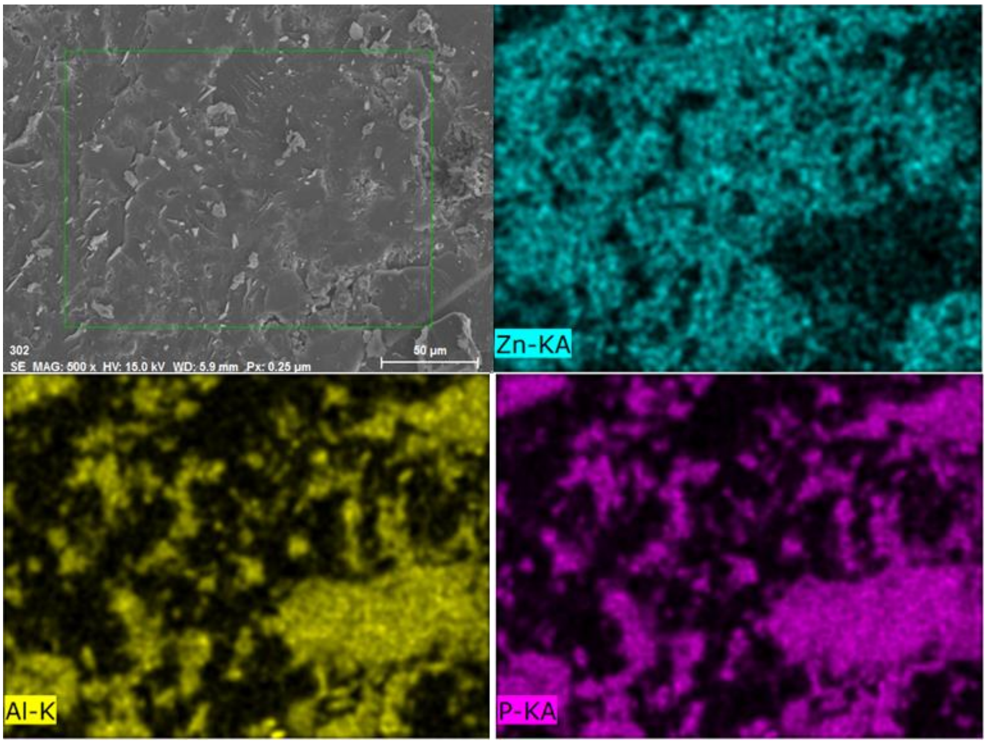

Supplementary Fig. 30. EDS of $(a_gZIF\text{-}62)_{0.5}(Na\text{-}deficient)_{0.5}$ – 1 min.

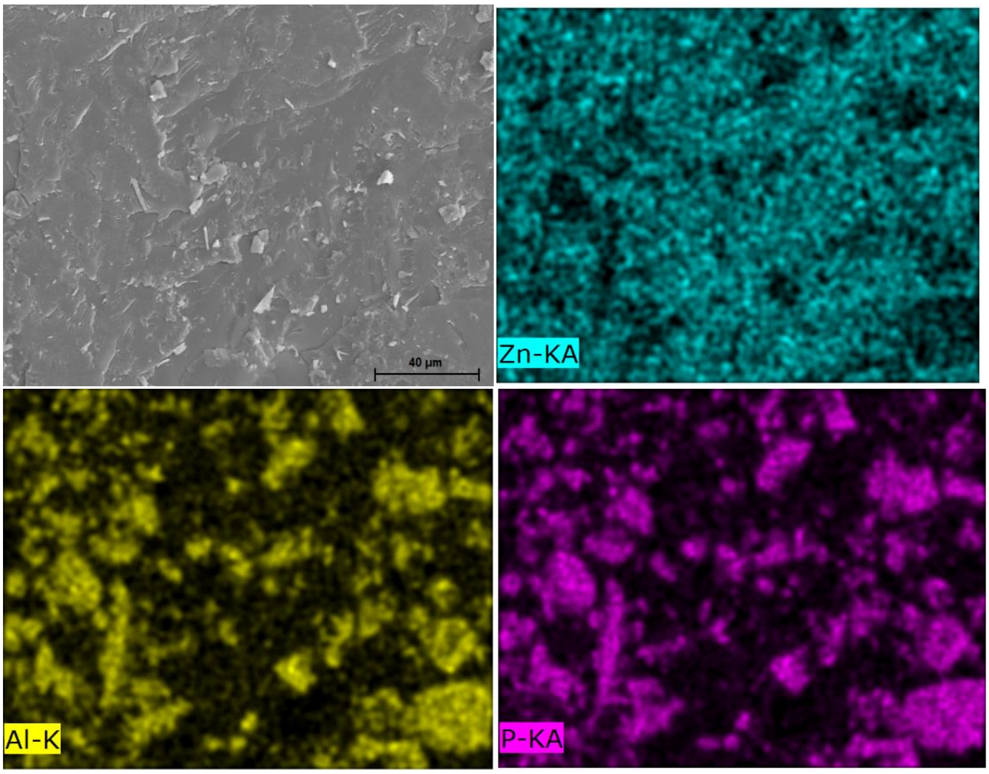

Supplementary Fig. 31. EDS of $(a_gZIF\text{-}62)_{0.5}(Na\text{-}deficient)_{0.5}$ – 30 min.

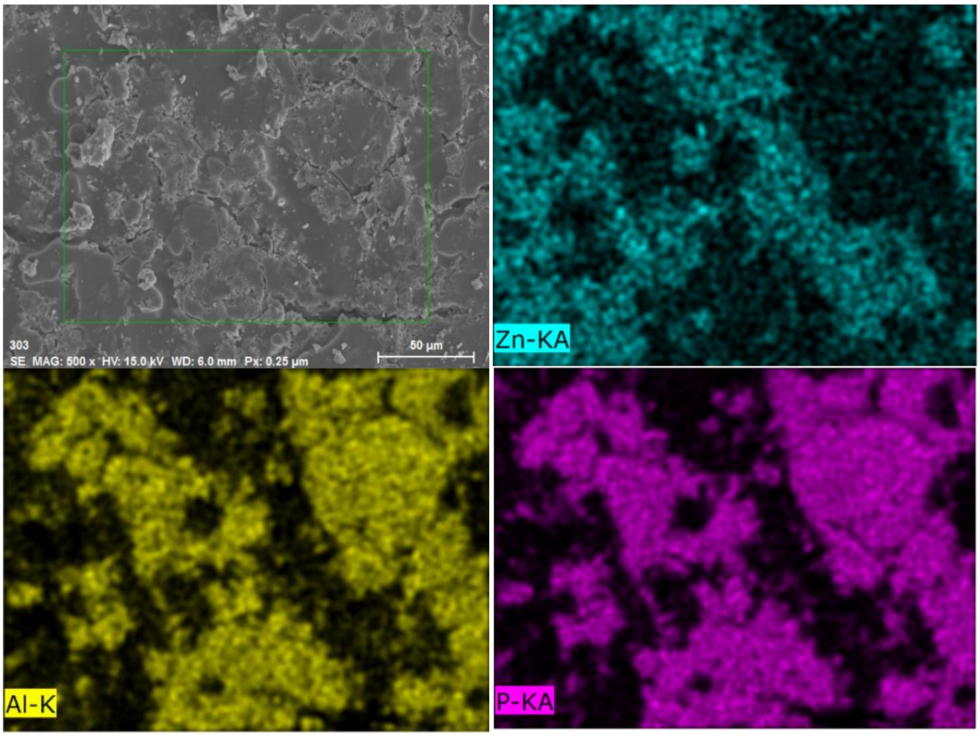

Supplementary Fig. 32. EDS of $(a_gZIF\text{-}62)_{0.5}(Al\text{-}rich)_{0.5}$ – 1 min.

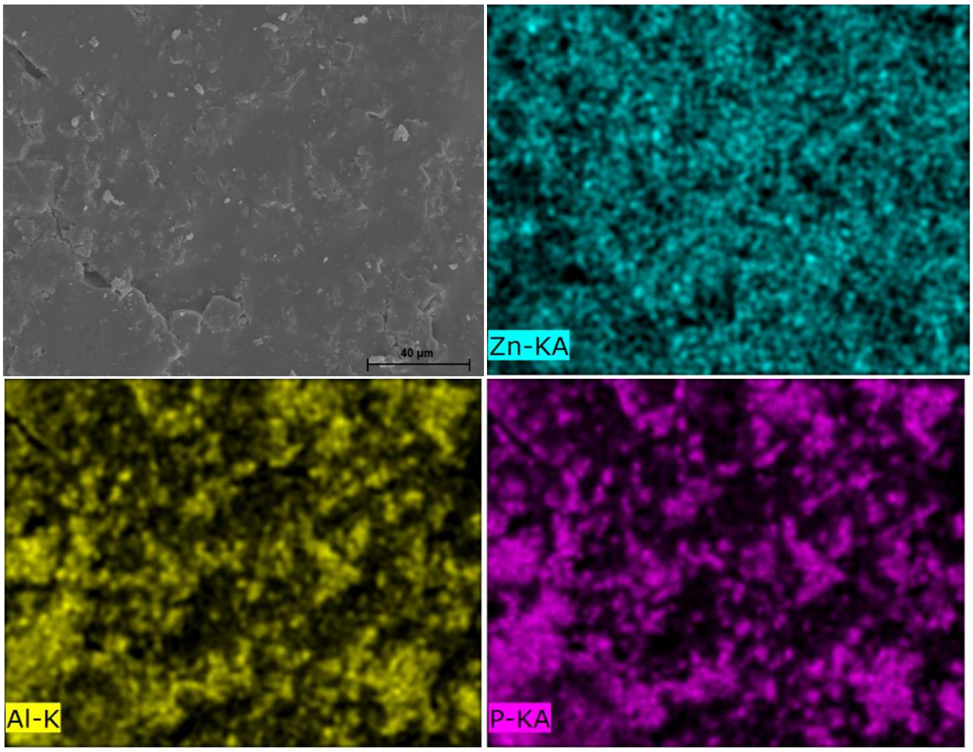

Supplementary Fig. 33. EDS of $(a_gZIF\text{-}62)_{0.5}(Al\text{-}rich)_{0.5}$ – 30 min.

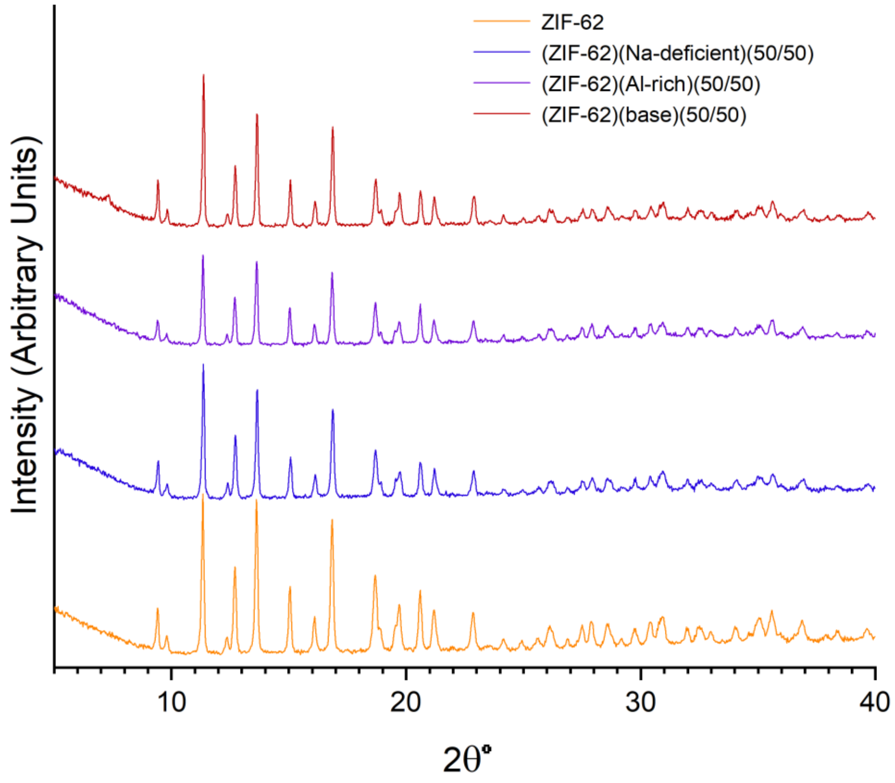

Supplementary Fig. 34. Powder X-ray diffraction patterns of the ball milled evacuated (ZIF-62)(Inorganic Glass)(50/50) mixtures and pure ZIF-62. An offset has been added to improve readability.

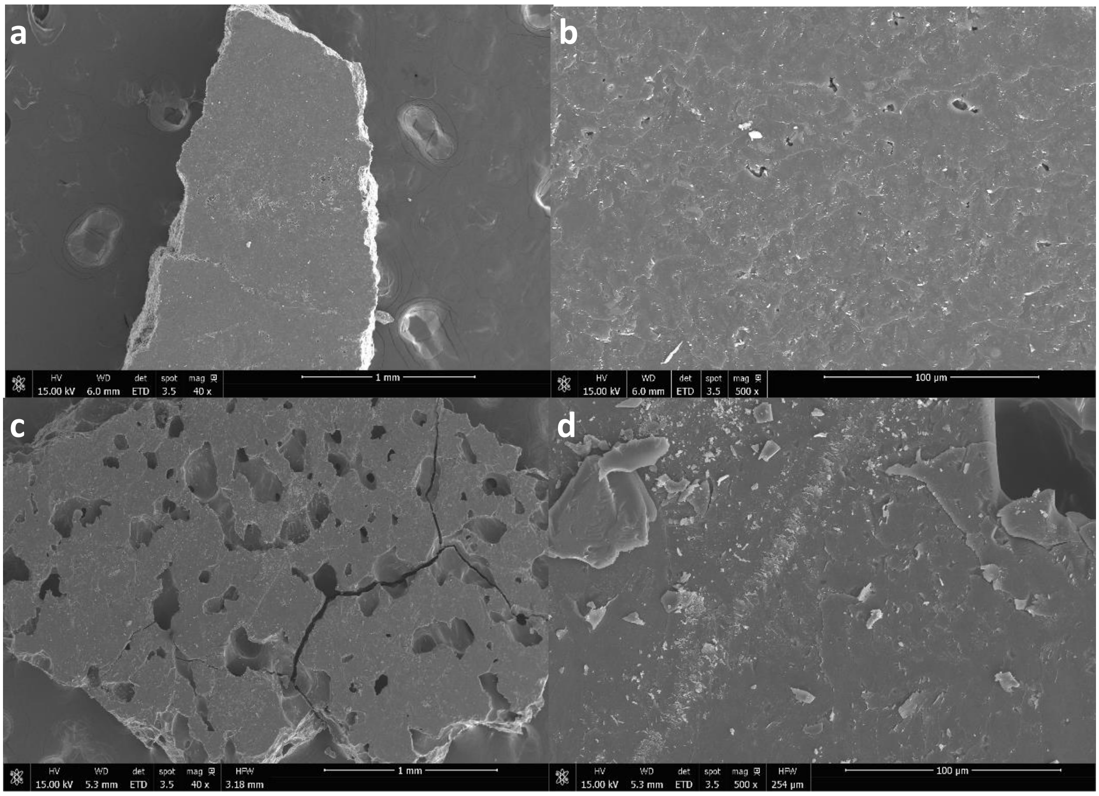

Supplementary Fig. 35. SEM of a) $(a_gZIF\text{-}62)_{0.5}(base)_{0.5}$ – 1 min 40 times magnification. b) $(a_gZIF\text{-}62)_{0.5}(base)_{0.5}$ – 1 min 500 times magnification. c) $(a_gZIF\text{-}62)_{0.5}(base)_{0.5}$ – 30 min 40 times magnification. d) $(a_gZIF\text{-}62)_{0.5}(base)_{0.5}$ – 30 min 500 times magnification.

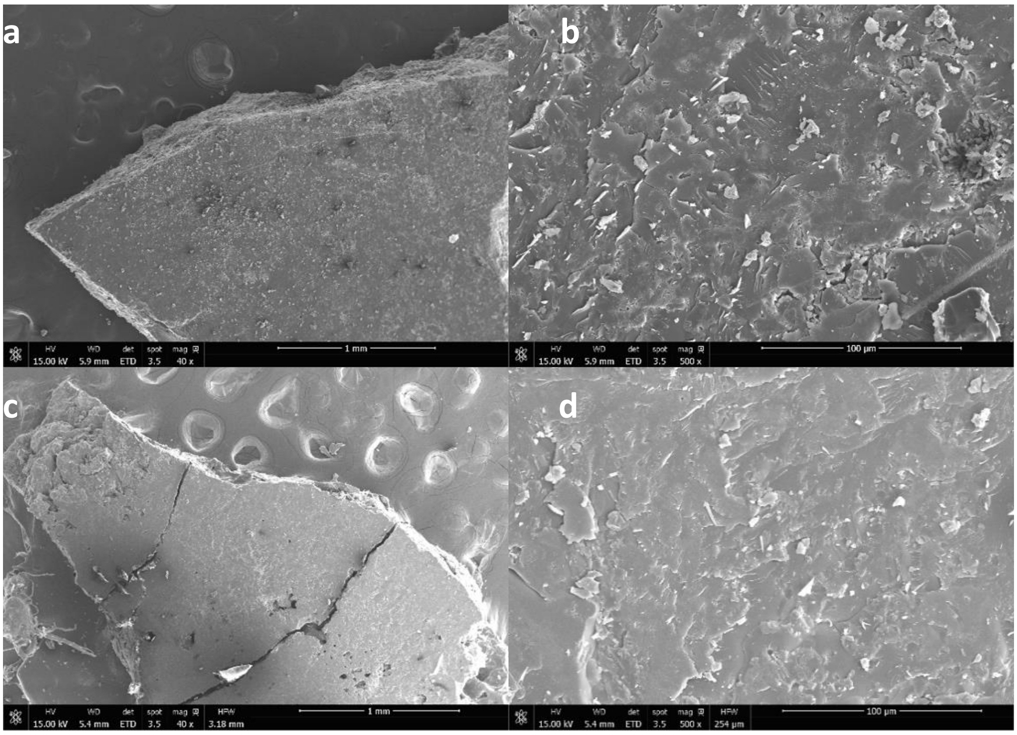

Supplementary Fig. 36. $(a_gZIF\text{-}62)_{0.5}(Na\text{-}deficient)_{0.5}$ – 1 min 40 times magnification. b) $(a_gZIF\text{-}62)_{0.5}(Na\text{-}deficient)_{0.5}$ – 1 min 500 times magnification. c) $(a_gZIF\text{-}62)_{0.5}(Na\text{-}deficient)_{0.5}$ – 30 min 40 times magnification. d) $(a_gZIF\text{-}62)_{0.5}(Na\text{-}deficient)_{0.5}$ – 30 min 500 times magnification.

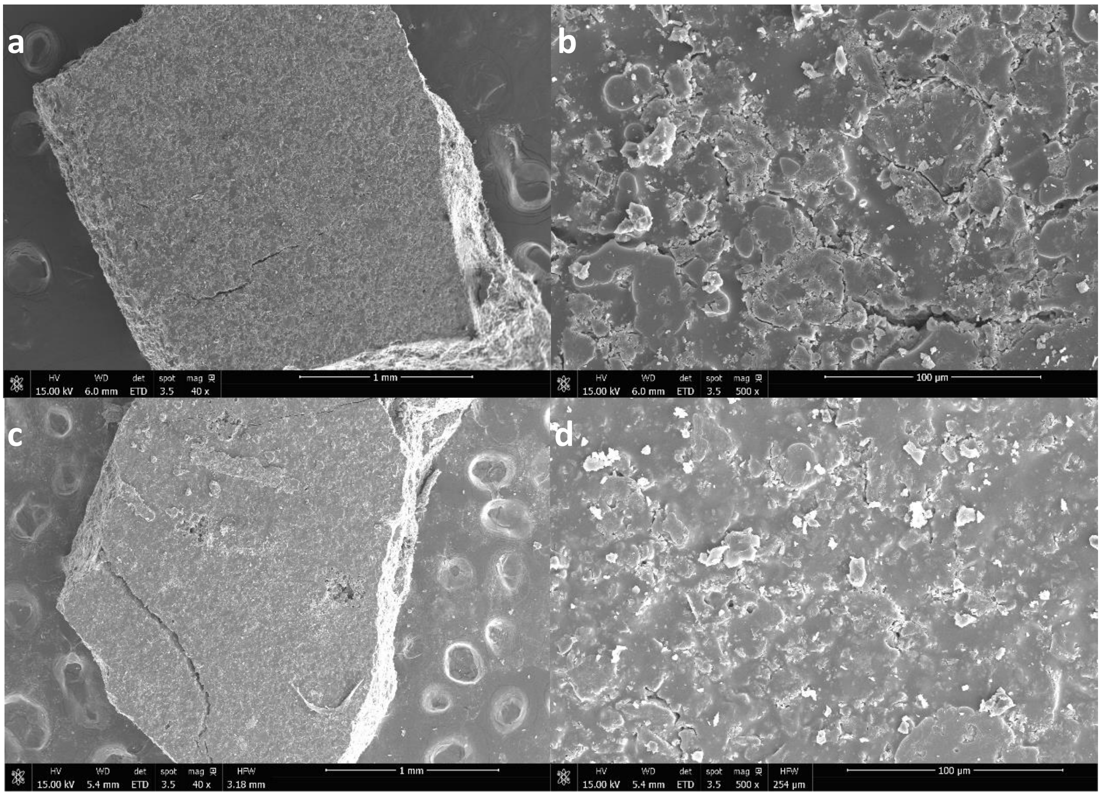

Supplementary Fig. 37. (a_gZIF-62)$_{0.5}$(Al-rich)$_{0.5}$ – 1 min 40 times magnification. b) (a_gZIF-62)$_{0.5}$(Al-rich)$_{0.5}$ – 1 min 500 times magnification. c) (a_gZIF-62)$_{0.5}$(Al-rich)$_{0.5}$ – 30 min 40 times magnification. d) (a_gZIF-62)$_{0.5}$(Al-rich)$_{0.5}$ – 30 min 500 times magnification.

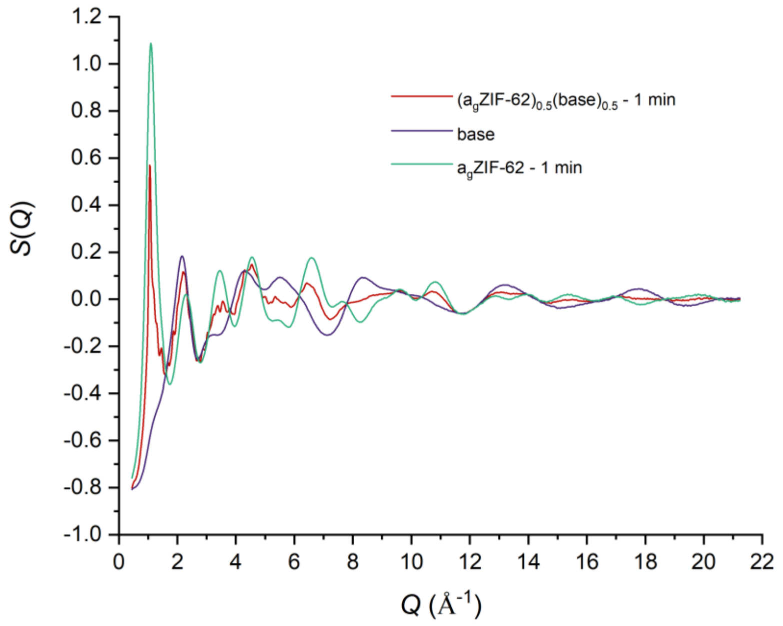

Supplementary Fig. 38. Structure factor *S*(*Q*) of $(a_gZIF\text{-}62)_{0.5}(base)_{0.5}$ – 1 min, $a_gZIF\text{-}62$ – 1 min and base.

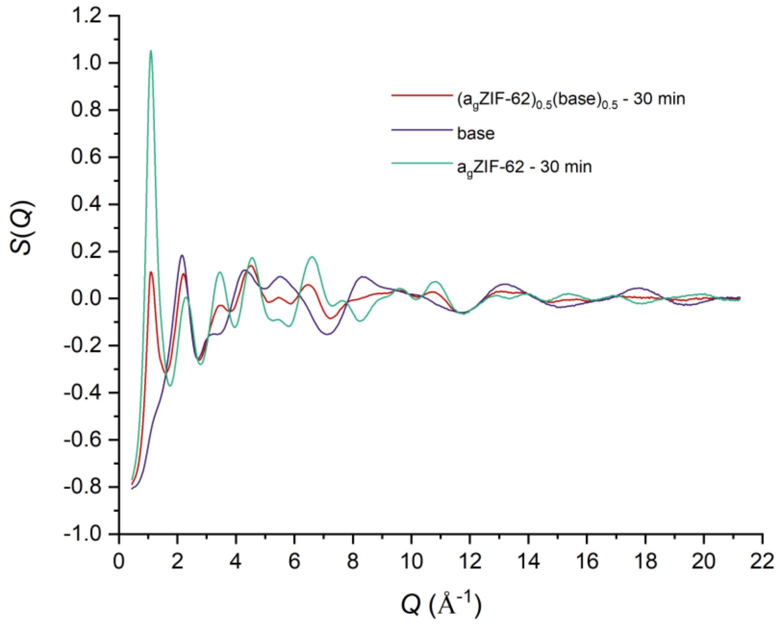

Supplementary Fig. 39. Structure factor *S*(*Q*) of $(a_gZIF\text{-}62)_{0.5}(base)_{0.5}$ –30 min, $a_gZIF\text{-}62$ – 30 min and base.

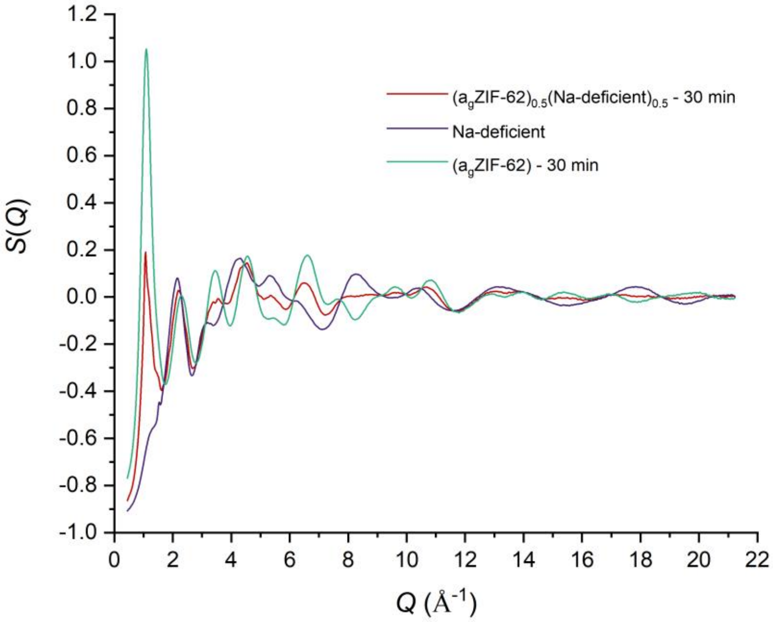

Supplementary Fig. 40. Structure factor *S*(*Q*) of $(a_gZIF\text{-}62)_{0.5}(Na\text{-}deficient)_{0.5}$ – 30 min, $a_gZIF\text{-}62$ – 30 min and Na deficient.

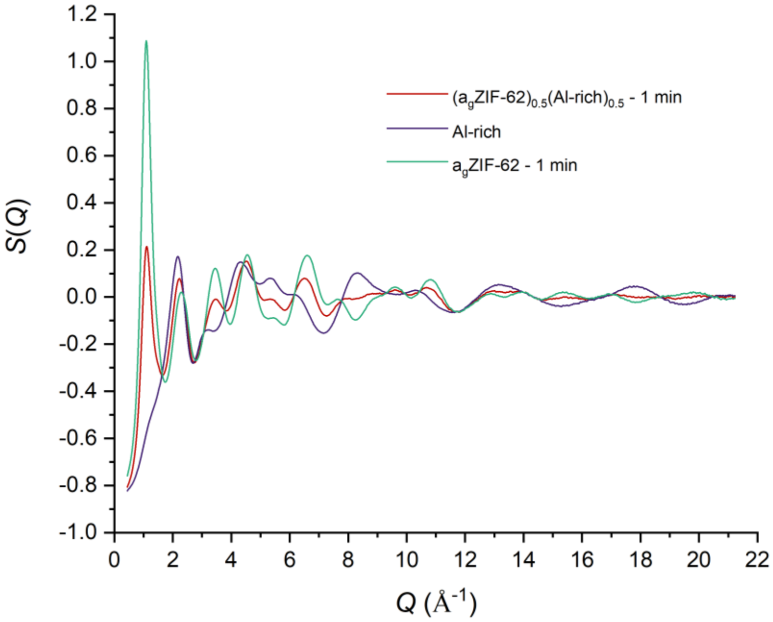

Supplementary Fig. 41. Structure factor S(Q) of $(a_gZIF\text{-}62)_{0.5}(Al\text{-}rich)_{0.5}$ – 1 min, $a_gZIF\text{-}62$ – 1 min and Al-rich.

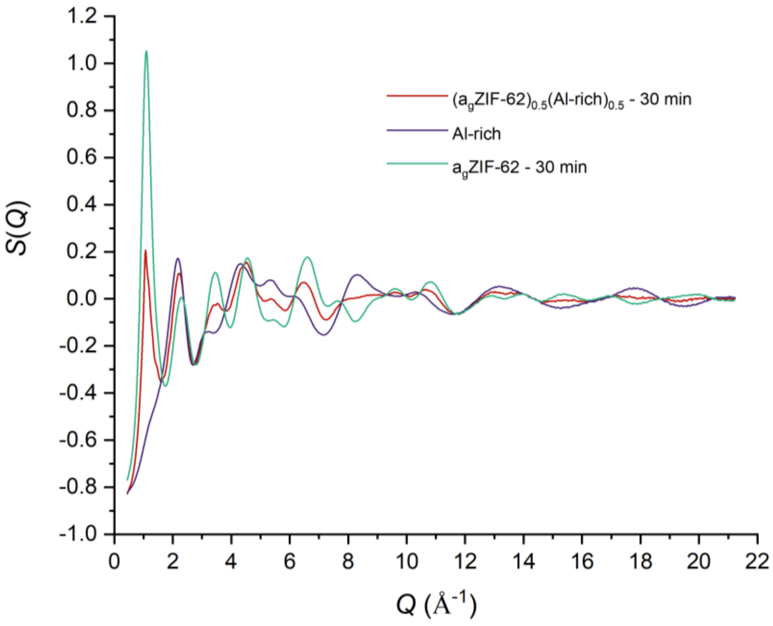

Supplementary Fig. 42. Structure factor S(Q) of $(a_gZIF\text{-}62)_{0.5}(Al\text{-}rich)_{0.5}$ – 30 min, a_gZIF-62 – 30 min and Al-rich.

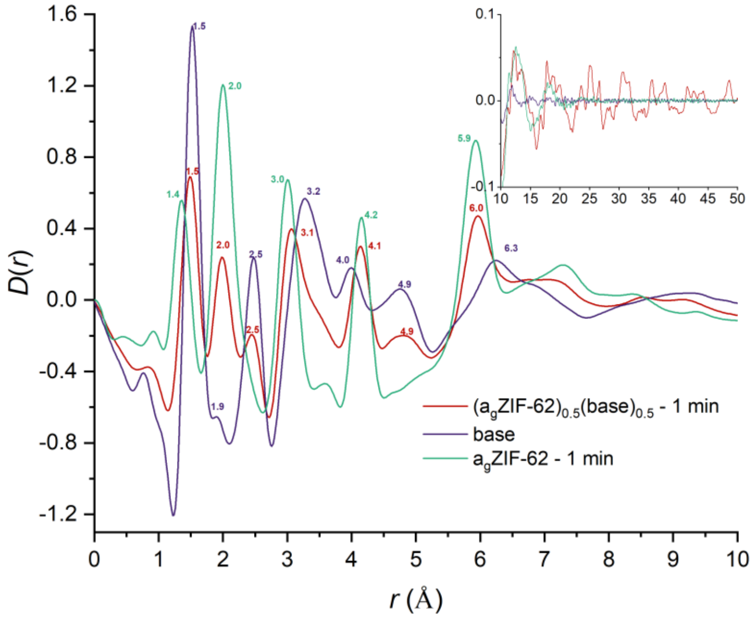

Supplementary Fig. 43. X-ray pair distribution functions *D*(*r*) of $(a_gZIF\text{-}62)_{0.5}(base)_{0.5}$ – 1 min, a_gZIF-62 –1min and base. Inset: *D*(*r*) plotted from 10-50 Å to show any long-range order.

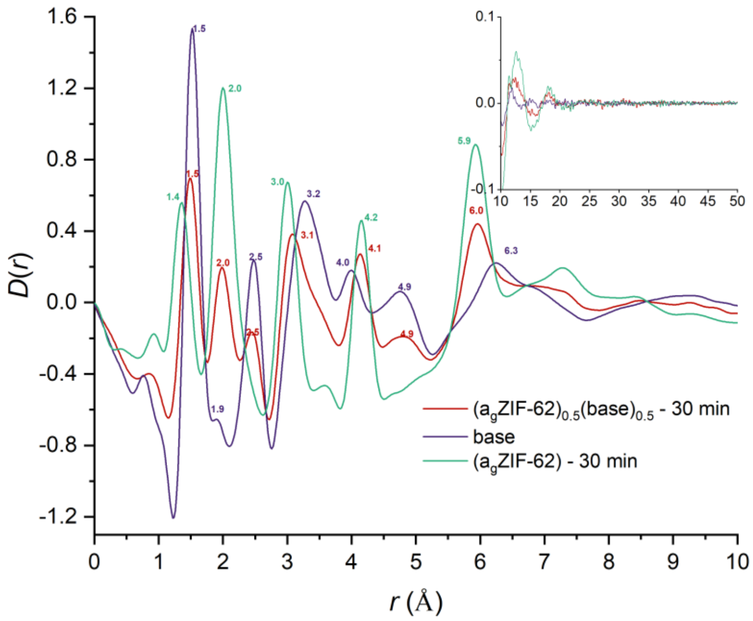

Supplementary Fig. 44. X-ray pair distribution functions $D(r)$ of $(a_gZIF\text{-}62)_{0.5}(base)_{0.5}$ – 30 min, a_gZIF-62 – 30min and base. Inset: $D(r)$ plotted from 10-50 Å to show any long-range order.

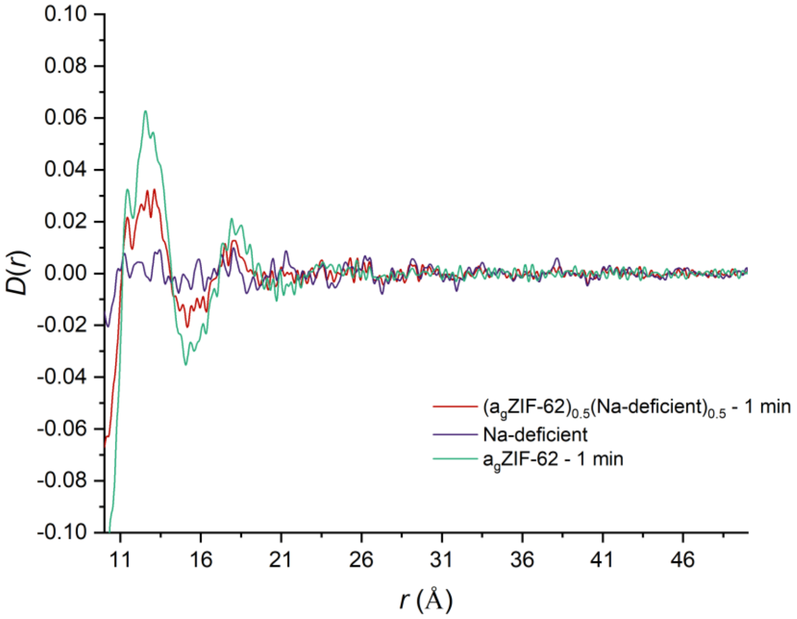

Supplementary Fig. 45. X-ray pair distribution functions $D(r)$ of $(a_gZIF\text{-}62)_{0.5}(Na\text{-}deficient)_{0.5}$ – 1 min, a_gZIF-62 – 1 min and Na-deficient to show any long range order.

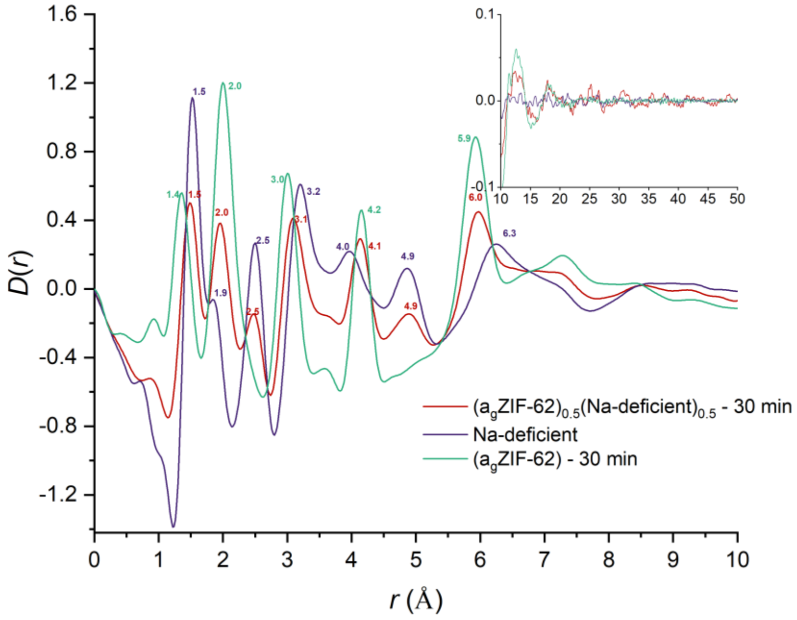

Supplementary Fig. 46. X-ray pair distribution functions $D(r)$ of $(a_gZIF\text{-}62)_{0.5}(Na\text{-}deficient)_{0.5}$ – 30 min, $a_gZIF\text{-}62$ – 30 min and Na-deficient. Inset: $D(r)$ plotted from 10-50 Å to show any long-range order..

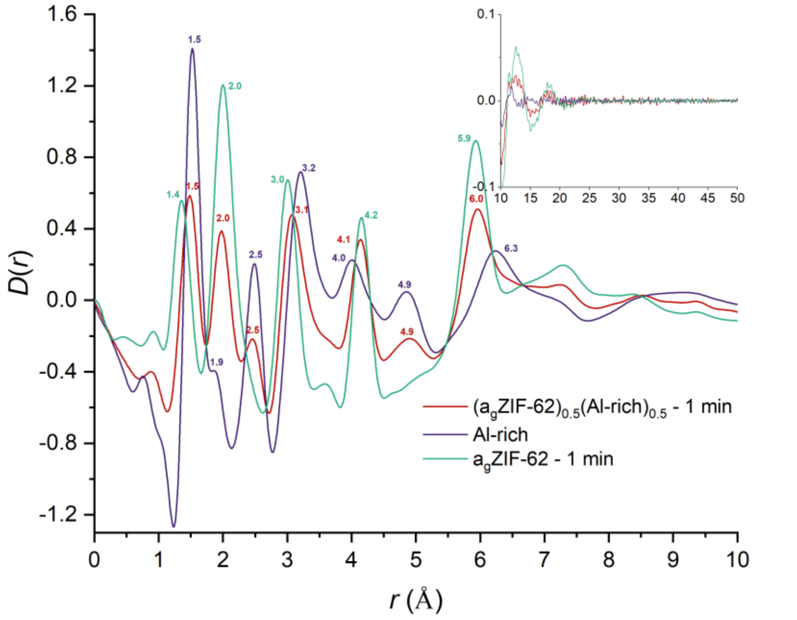

Supplementary Fig. 47. X-ray pair distribution functions $D(r)$ of $(a_gZIF\text{-}62)_{0.5}(Al\text{-}rich)_{0.5}$ – 1 min, $a_gZIF\text{-}62$ – 1min and Al-rich. Inset: $D(r)$ plotted from 10-50 Å to show any long-range order.

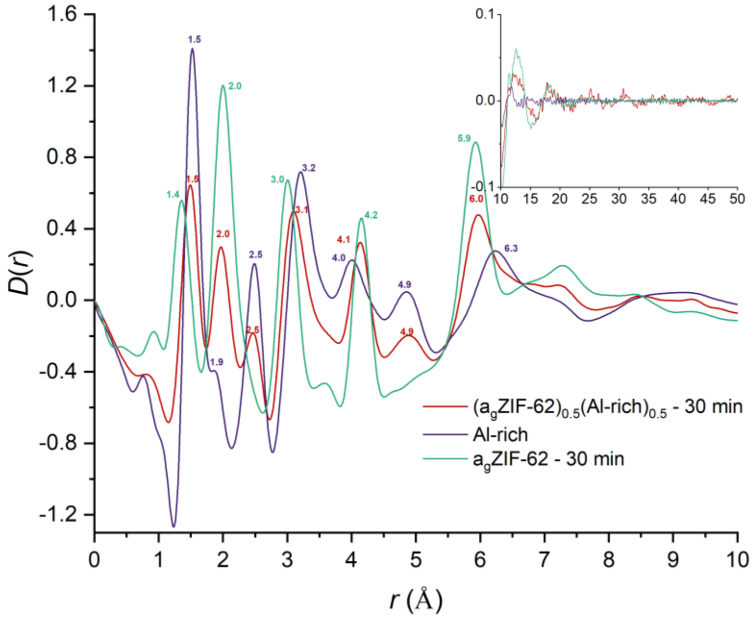

Supplementary Fig. 48. X-ray pair distribution functions $D(r)$ of $(a_gZIF\text{-}62)_{0.5}(Al\text{-}rich)_{0.5}$ – 30 min, a_gZIF-62 – 30 min and Al-rich. Inset: $D(r)$ plotted from 10-50 Å to show any long-range order.

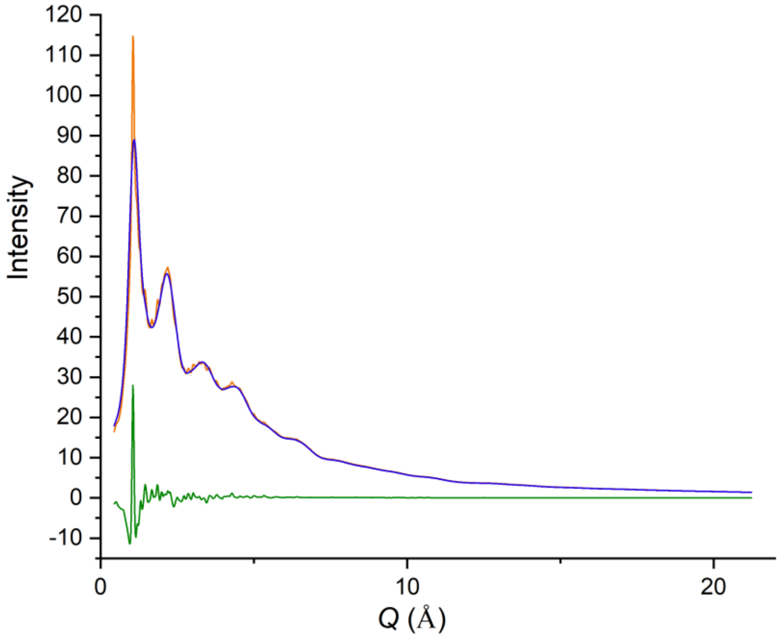

Supplementary Fig. 49. (a) Experimental (orange) vs calculated (blue) total scattering and difference (green) of $(a_gZIF\text{-}62)_{0.5}(base)_{0.5}$ – 1 min.

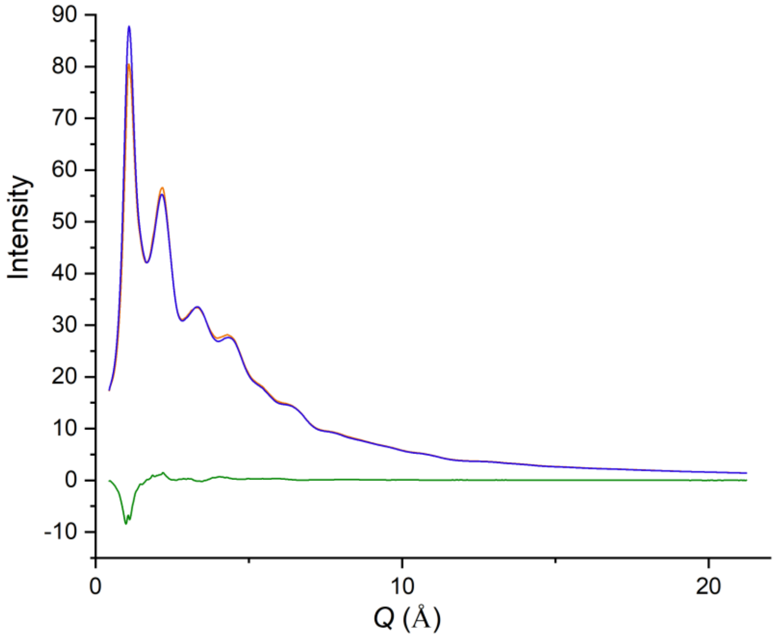

Supplementary Fig. 50. (a) Experimental (orange) vs calculated (blue) total scattering and difference (green) of $(a_gZIF\text{-}62)_{0.5}(base)_{0.5}$ – 30 min.

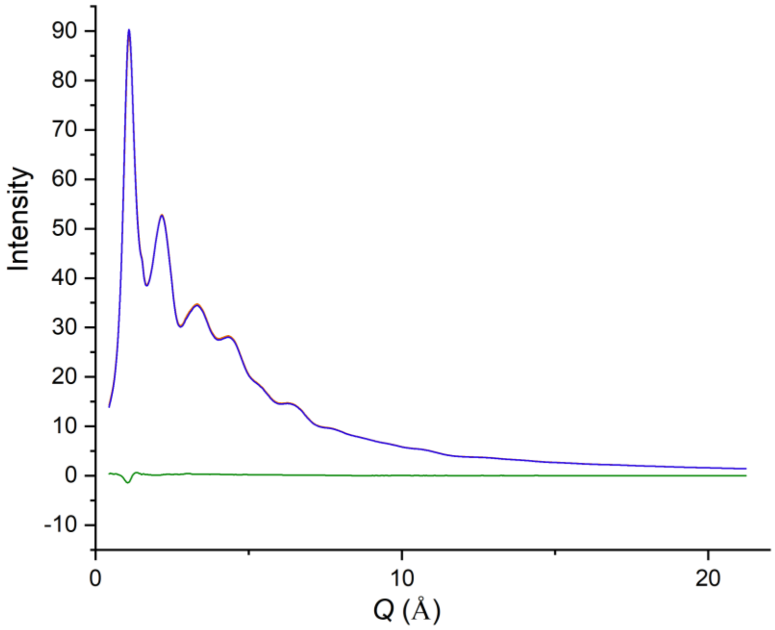

Supplementary Fig. 51. (a) Experimental (orange) vs calculated (blue) total scattering and difference (green) of $(a_g\text{ZIF-62})_{0.5}(\text{Na-deficient})_{0.5}$ – 1 min.

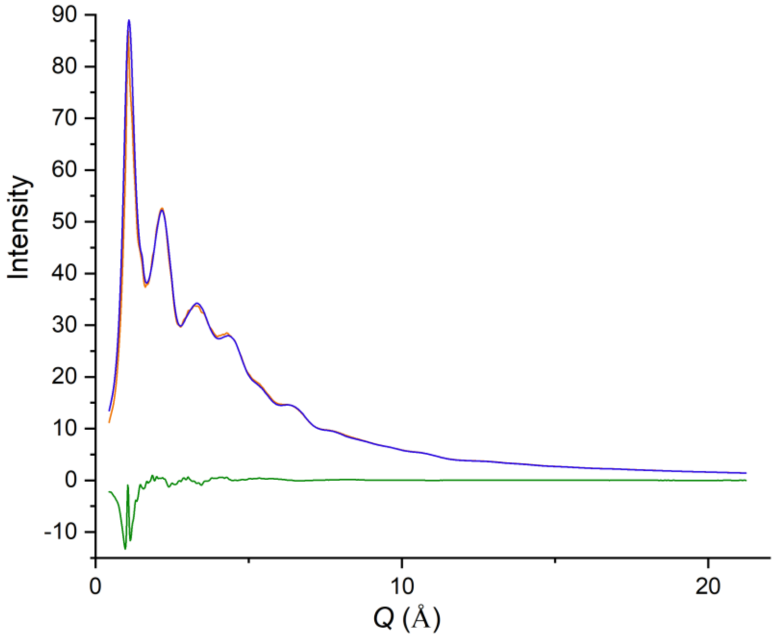

Supplementary Fig. 52. (a) Experimental (orange) vs calculated (blue) total scattering and difference (green) of $(a_g\text{ZIF-62})_{0.5}(\text{Na-deficient})_{0.5}$ – 30 min.

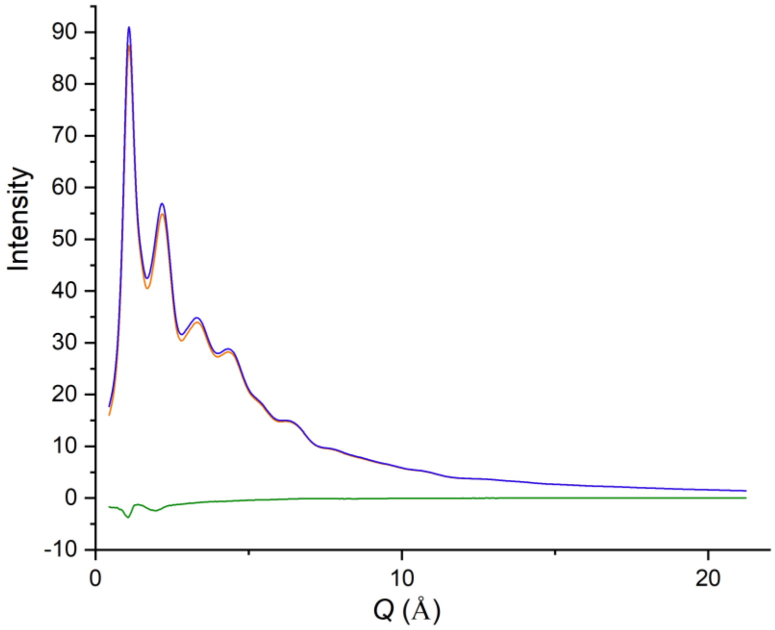

Supplementary Fig. 53. (a) Experimental (orange) vs calculated (blue) total scattering and difference (green) of (a_gZIF-62)$_{0.5}$(Al-rich)$_{0.5}$ – 1 min.

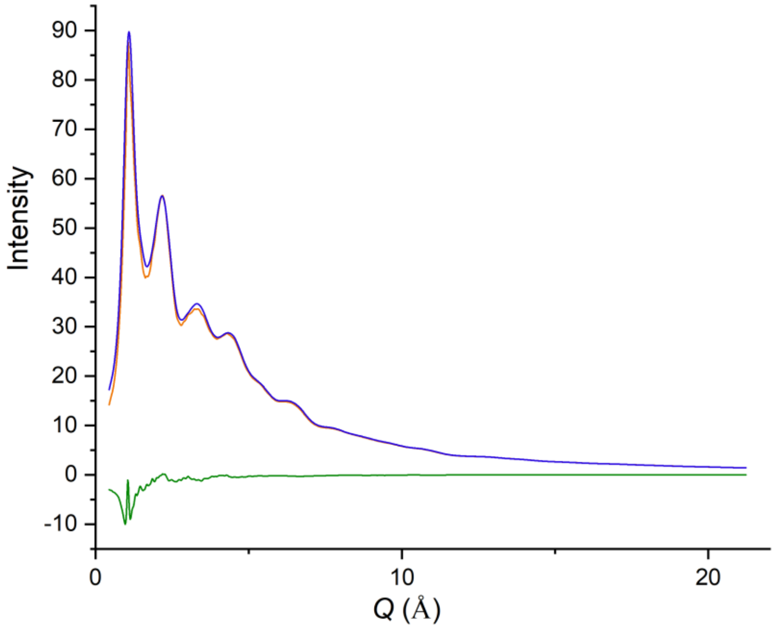

Supplementary Fig. 54. (a) Experimental (orange) vs calculated (blue) total scattering and difference (green) of (a_gZIF-62)$_{0.5}$(Al-rich)$_{0.5}$ – 30 min.

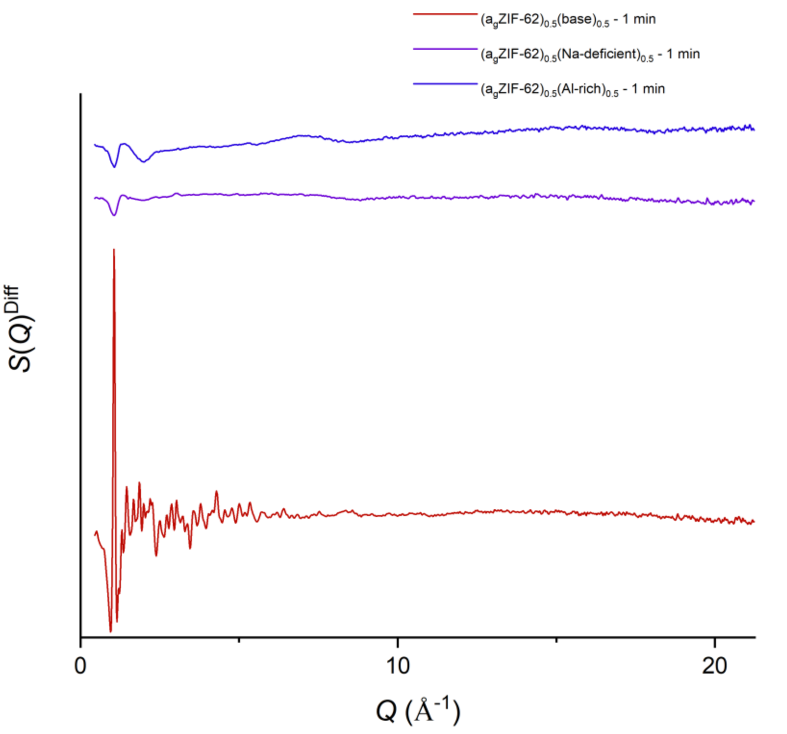

Supplementary Fig. 55. $S(Q)^{Diff}$ for (a_gZIF-62)$_{0.5}$(Inorganic Glass)$_{0.5}$ – 1 min samples. An offset has been added to improve readability.

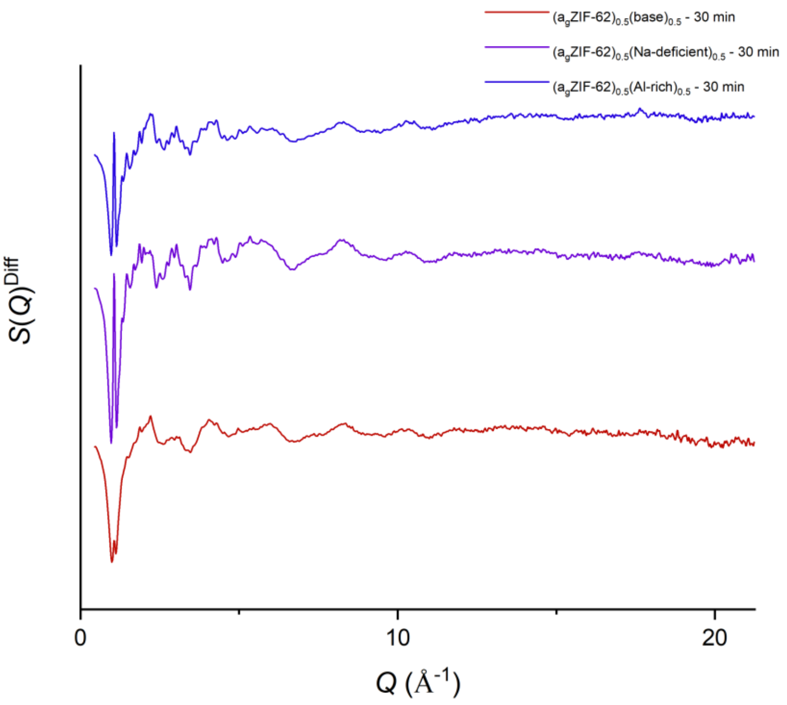

Supplementary Fig. 56. $S(Q)^{Diff}$ for (a_gZIF-62)$_{0.5}$(inorganic)$_{0.5}$ – 30 min samples. An offset has been added to improve readability.

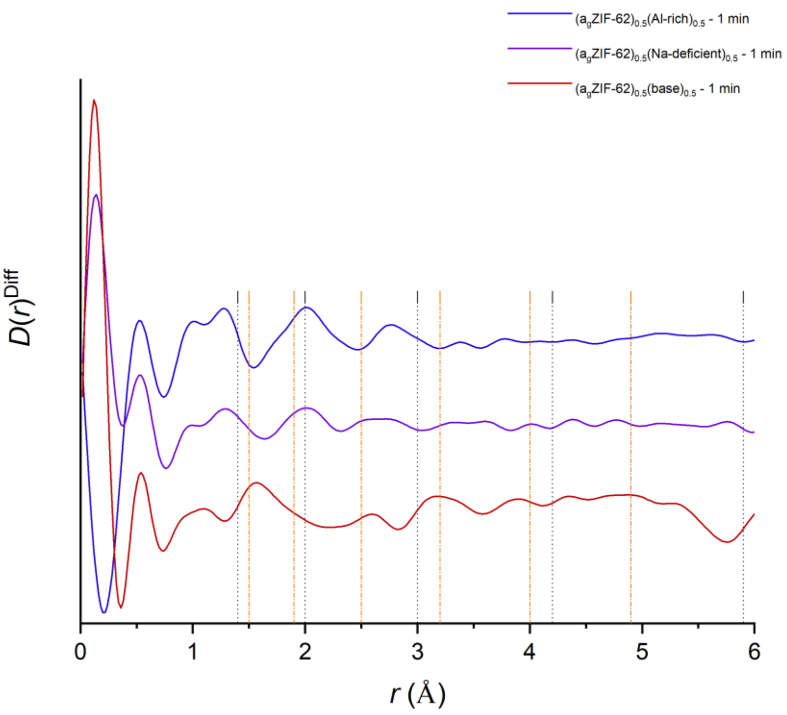

Supplementary Fig. 57. $D(r)^{Diff}$ of the ($a_gZIF-62)_{0.5}$(Inorganic Glass$)_{0.5}$ – 1 min samples showing the short range structure. Droplines indicate correlations expected from the $a_gZIF-62$ (black) and Inorganic Glass (orange). An offset has been added to improve readability.

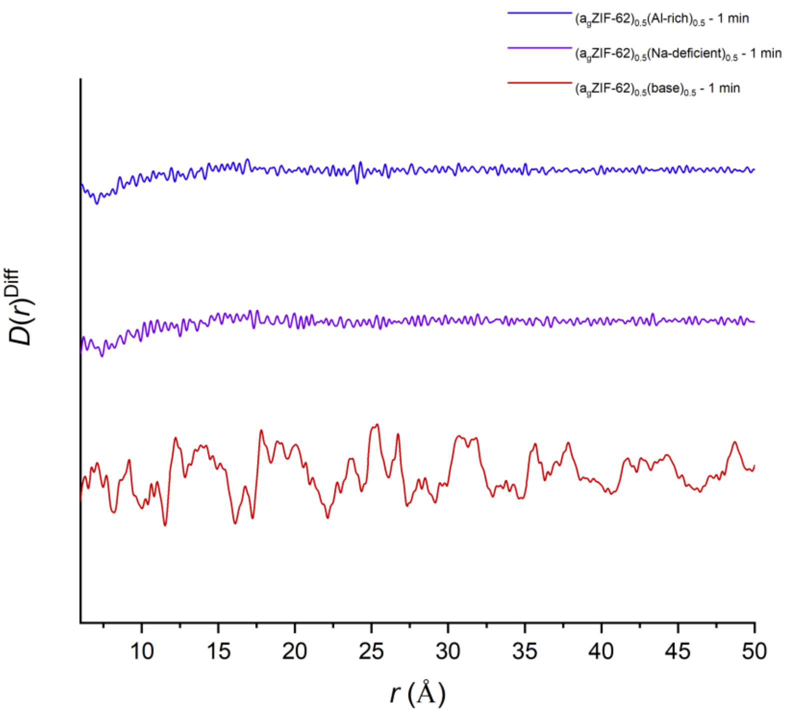

Supplementary Fig. 58. Extended $D(r)^{Diff}$ of the ($a_gZIF-62)_{0.5}$(Inorganic Glass$)_{0.5}$ – 1 min samples. An offset has been added to improve readability.

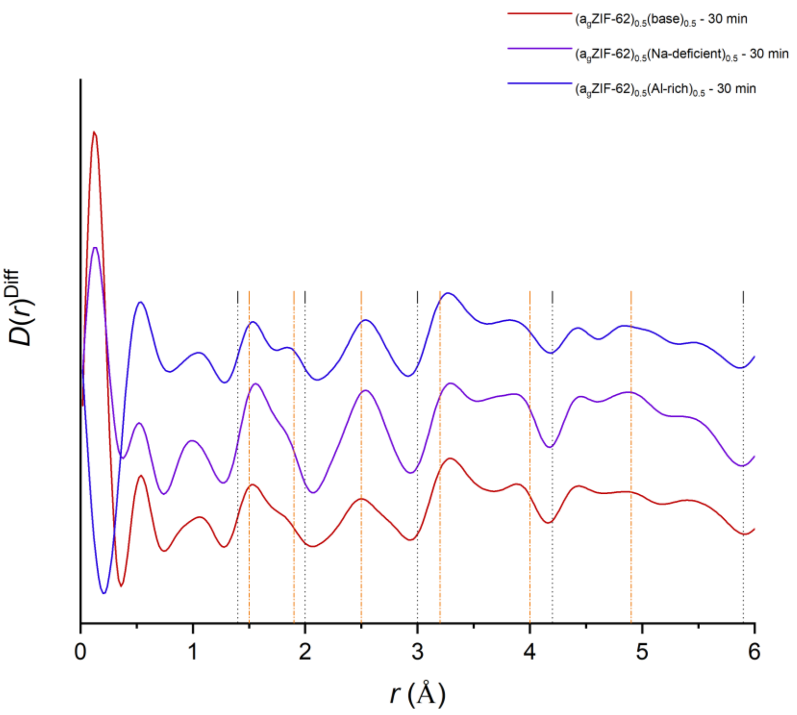

Supplementary Fig. 59. $D(r)^{\mathrm{Diff}}$ of the $(a_gZIF\text{-}62)_{0.5}(Inorganic\ Glass)_{0.5}$ – 30 min samples showing the short range structure. Droplines indicate correlations expected from the a_gZIF-62 (black) and Inorganic Glass (orange). An offset has been added to improve readability.

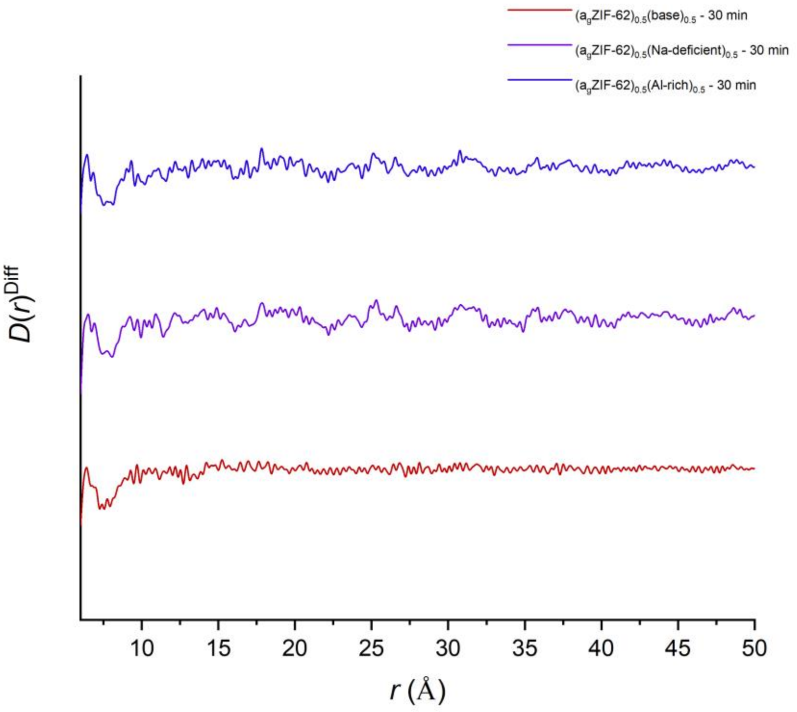

Supplementary Fig. 60. Extended $D(r)^{\mathrm{Diff}}$ of the the $(a_gZIF\text{-}62)_{0.5}(Inorganic\ Glass)_{0.5}$ – 30 min samples. An offset has been added to improve readability.

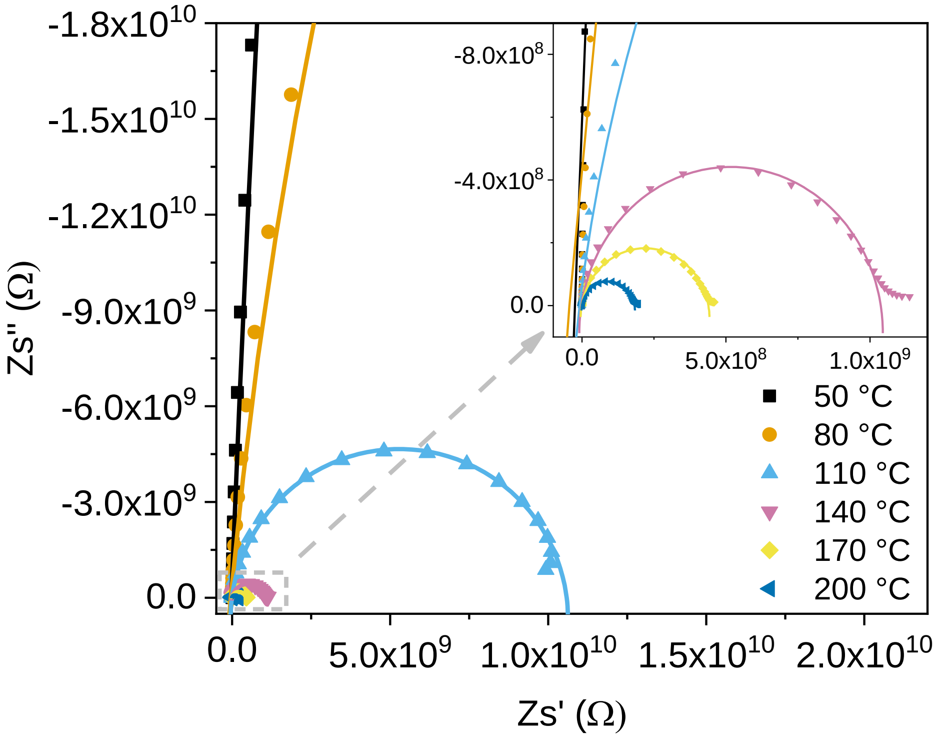

Supplementary Fig. 61. Nyquist Plot of $(a_gZIF\text{-}62)_{0.5}(Na\text{-deficient})_{0.5}$ – 1 min composite.

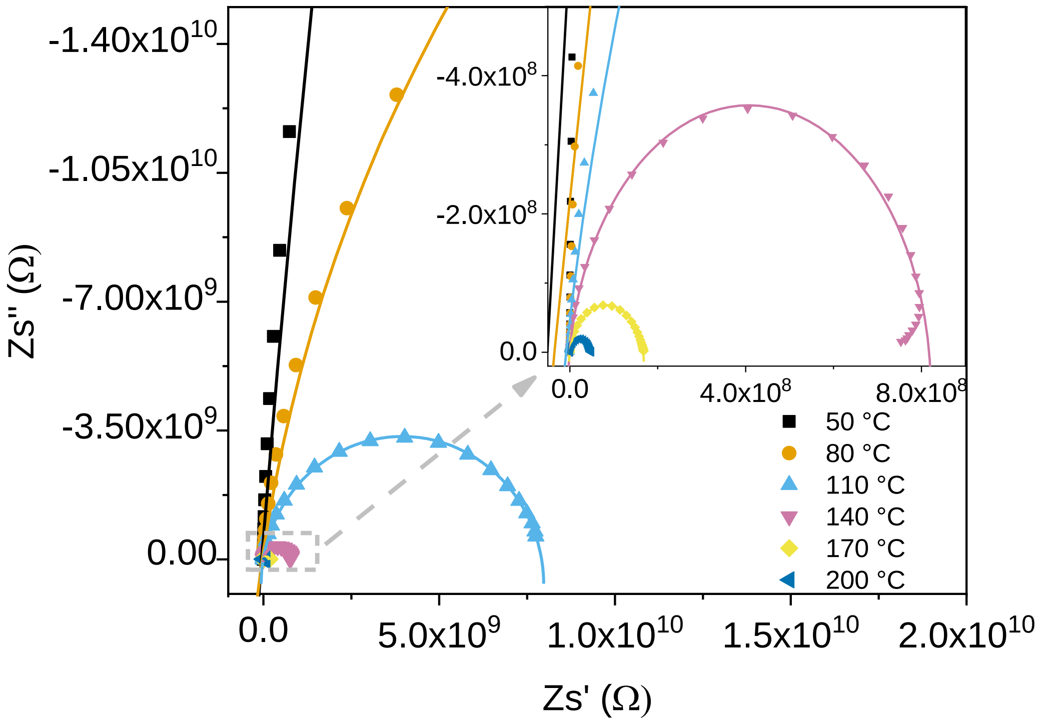

Supplementary Fig. 62. Nyquist Plot of $(a_gZIF\text{-}62)_{0.5}(Na\text{-deficient})_{0.5}$ – 30 min composite.

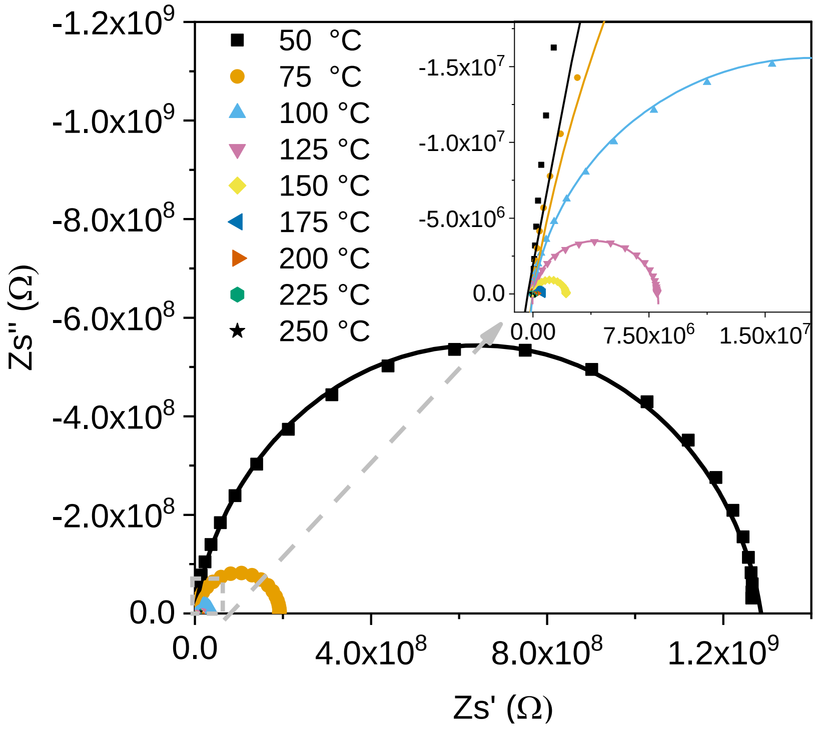

Supplementary Fig. 63. Nyquist Plot of Na-deficient inorganic sample.

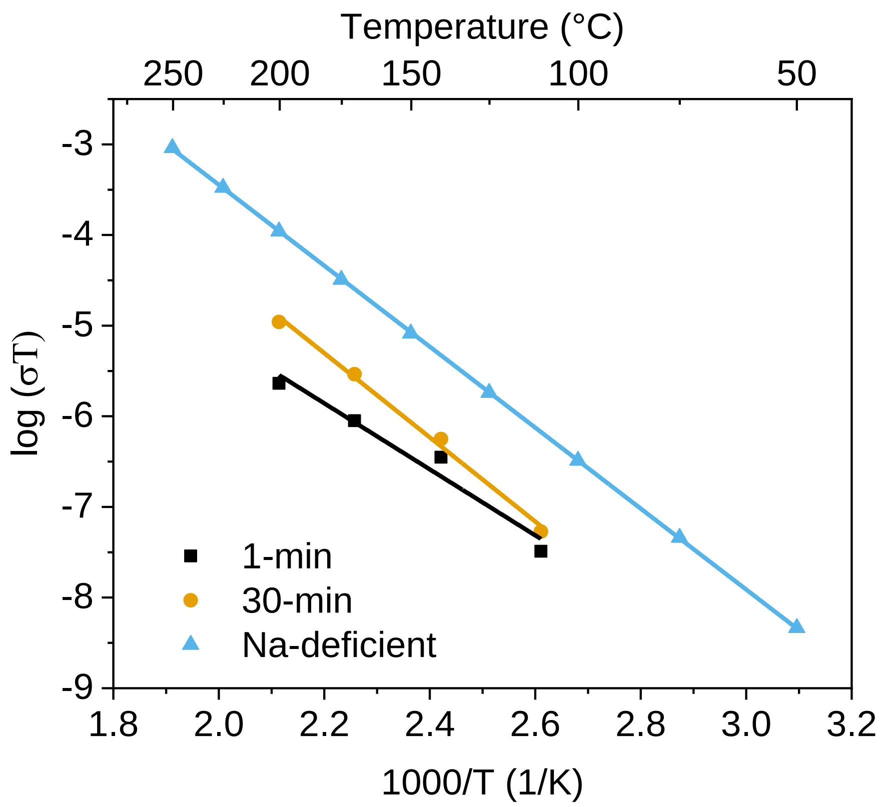

Supplementary Fig. 64. Arrhenius plots of $(a_gZIF\text{-}62)_{0.5}(Na\text{-}deficient)_{0.5}$ – 1 min, $(a_gZIF\text{-}62)_{0.5}(Na\text{-}deficient)_{0.5}$ – 30 min and Na-deficient inorganic samples.

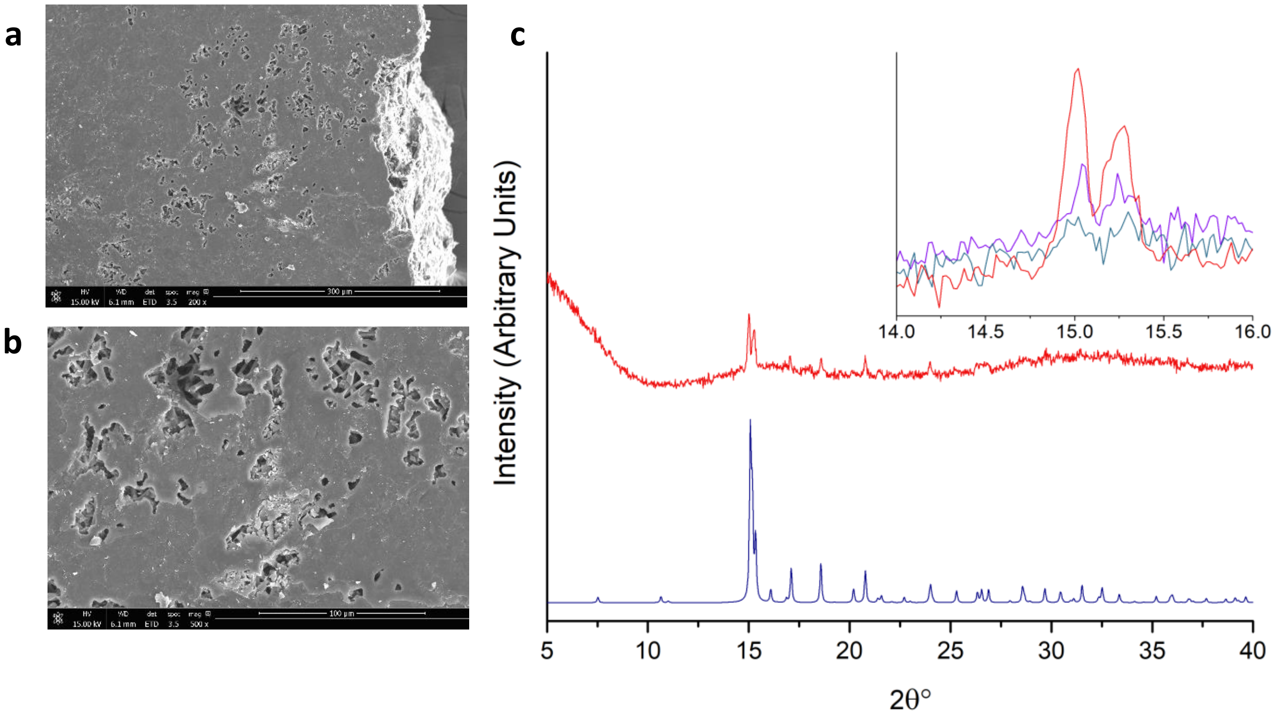

Supplementary Fig. 65. SEM Images of $(a_gZIF\text{-}62)_{0.5}(base)_{0.5}$ - 1 min at a) 200 times magnification and b) 500 times magnification. c) XRD pattern of $(a_gZIF\text{-}62)_{0.5}(base)_{0.5}$ - 1 min (red) and ZIF-zni reference pattern (blue)[9]. An offset has been added to improve readability. Inset XRD pattern of i) $(a_gZIF\text{-}62)_{0.5}(base)_{0.5}$ - 1 min (red) ii) $(a_gZIF\text{-}62)_{0.5}(Na\text{-}deficient)_{0.5}$ - 30 min (purple) iii) $(a_gZIF\text{-}62)_{0.5}(Al\text{-}rich)_{0.5}$ - 30 min (cyan).

Supplementary Tables

Supplementary Table 1. Glass transition temperatures (T_g), analysed (from EDS) compositions of the (**1−*x***)([Na_2O]$_z$[P_2O_5])-***x***([$AlO_{3/2}$][AlF_3]$_y$) inorganic glass series.

Inorganic Glass	T_g (°C)	Analysed (mol%)			
		**P_2O_5**	**Na_2O**	**Al_2O_3**	**AlF_3**
**base**	372	31.6 ± 0.2	52.0 ± 0.1	7.1 ± 0.1	9.4 ± 0.1
**Na-deficient**	414	38.7 ± 0.2	33.3 ± 0.1	7.1 ± 0.1	20.9 ± 0.2
**Al-rich**	449	28.3 ± 0.2	47.2 ± 0.1	13.6 ± 0.1	11.0 ± 0.1

Supplementary Table 2. Roughness profile parameters of composites heat treated for 1 and 30 minutes. Sample is specified by the inorganic composition: 'base – 1 min' corresponds to (a_gZIF-62)$_{0.5}$(base)$_{0.5}$ – 1 min composition. The error is assumed to be determined by instrument laser wavelength and pinhole size, see experimental methods.

P_c: Mean height of the primary profile elements			
**Sample**	**base**	**Na-deficient**	**Al-rich**
**1 min**	4.787	6.432	13.333
**30 min**	4.373	2.938	9.681
P_a: Arithmetical mean deviation of the primary profile			
**Sample**	**base**	**Na-deficient**	**Al-rich**
**1 min**	1.183	0.609	2.536
**30 min**	0.79	0.666	2.422
P_z: Maximum height of the primary profile			
**Sample**	**base**	**Na-deficient**	**Al-rich**
**1 min**	10.371	12.052	21.34
**30 min**	7.677	4.2	13.974

For the following Supplementary Tables 3 – 9, please refer to Supplementary Supplementary Fig. 10 for the labelling of imidazole and benzimidazole molecules (Benz-1, Imid-A, etc.).

Supplementary Table 3. Liquid ^{1}H NMR chemical shifts of crystalline and amorphous pure ZIF-62 controls (1 and 30 min heat treatments).

	Chemical Shift (ppm)				
**Sample**	Benz-1	Imid-A	Benz-2	Imid-B	Benz-3
**ZIF-62**	7.57	7.58	7.84	9.00	9.52
**a_gZIF-62 – 1 min**	7.53	7.56	7.81	8.97	9.50
**a_gZIF-62 – 30 min**	7.53	7.55	7.81	8.97	9.50

Supplementary Table 4. Liquid ^{1}H NMR chemical shifts of composites heat treated for 1 min.

	Chemical Shift (ppm)				
**Composite**	Benz-1	Imid-A	Benz-2	Imid-B	Benz-3
**a_gZIF-62 – 1 min**	7.53	7.56	7.81	8.97	9.50
**$(a_g\text{ZIF-62})_{0.5}(\text{base})_{0.5}$ – 1 min**	7.52	7.54	7.81	8.95	9.48
**$(a_g\text{ZIF-62})_{0.5}(\text{Na-deficient})_{0.5}$ – 1 min**	7.53	7.56	7.81	8.98	9.50
**$(a_g\text{ZIF-62})_{0.5}(\text{Al-rich})_{0.5}$ – 1 min**	7.54	7.56	7.82	8.98	9.50

Supplementary Table 5. Liquid ^{1}H NMR chemical shifts of composites heat treated for 30 min.

	Chemical Shift (ppm)				
**Composite**	Benz-1	Imid-A	Benz-2	Imid-B	Benz-3
**a_gZIF-62 – 30 min**	7.53	7.55	7.81	8.97	9.50
**$(a_g\text{ZIF-62})_{0.5}(\text{base})_{0.5}$ – 30 min**	7.54	7.56	7.82	8.98	9.50
**$(a_g\text{ZIF-62})_{0.5}(\text{Na-deficient})_{0.5}$ – 30 min**	7.53	7.56	7.81	8.97	9.50
**$(a_g\text{ZIF-62})_{0.5}(\text{Al-rich})_{0.5}$ – 30 min**	7.53	7.56	7.81	8.97	9.50

Supplementary Table 6. Liquid ^{1}H NMR J-couplings of crystalline and amorphous pure ZIF-62 controls (1 and 30 min heat treatments).

	J-coupling (Hz)					
**Composite**	Benz-1	Benz-1	Imid-A	Benz-2	Benz-2	Imid-B
**ZIF-62**	3.16	6.21	1.33	3.12	6.25	1.32
**a_gZIF-62 – 1 min**	3.16	6.22	1.30	3.11	6.25	1.28
**a_gZIF-62 – 30 min**	3.18	6.23	1.25	3.15	6.20	1.20

Supplementary Table 7. Liquid ^1H NMR J-couplings of composites heat treated for 1 min.

Composite	J-coupling (Hz)					
	Benz-1	Benz-1	Imid-A	Benz-2	Benz-2	Imid-B
**a_gZIF-62 – 1 min**	3.16	6.22	1.30	3.11	6.25	1.28
**(a_gZIF-62)$_{0.5}$(base)$_{0.5}$ – 1 min**	3.15	6.25	1.25	3.13	6.23	1.23
**(a_gZIF-62)$_{0.5}$(Na-deficient)$_{0.5}$ – 1 min**	3.15	6.22	1.31	3.12	6.24	1.29
**(a_gZIF-62)$_{0.5}$(Al-rich)$_{0.5}$ – 1 min**	3.15	6.22	1.31	3.10	6.26	1.29

Supplementary Table 8. Liquid ^1H NMR J-couplings of composites heat treated for 30 min.

Composite	J-coupling (Hz)					
	Benz-1	Benz-1	Imid-A	Benz-2	Benz-2	Imid-B
**a_gZIF-62 – 30 min**	3.18	6.23	1.25	3.15	6.20	1.20
**(a_gZIF-62)$_{0.5}$(base)$_{0.5}$ – 30 min**	3.15	6.20	1.30	3.13	6.23	1.27
**(a_gZIF-62)$_{0.5}$(Na-deficient)$_{0.5}$ – 30 min**	3.15	6.25	1.30	3.13	6.23	1.26
**(a_gZIF-62)$_{0.5}$(Al-rich)$_{0.5}$ – 30 min**	3.15	6.20	1.30	3.13	6.23	1.24

Supplementary Table 9. [bIm]/[bIm+Im] ratio from ^1H NMR integrals of singlet (9.5 ppm) and triplet (9.0 ppm), respectively.

Pure ZIF-62 Controls		
**ZIF-62**	**a_gZIF-62 – 1 min**	**a_gZIF-62 – 30 min**
0.118	0.117	0.117
	**Treatment Time**	
**Composite**	1 min	30 min
**(a_gZIF-62)$_{0.5}$(base)$_{0.5}$**	0.118	0.120
**(a_gZIF-62)$_{0.5}$(Na-deficient)$_{0.5}$**	0.116	0.120
**(a_gZIF-62)$_{0.5}$(Al-rich)$_{0.5}$**	0.116	0.120

Supplementary Table 10. Densities (ρ), as measured by the Archimedean method, of the $(\mathbf{1-x})([Na_2O]_z[P_2O_5])$-$\mathbf{x}([AlO_{3/2}][AlF_3]_y)$ inorganic glass series. Error was calculated at 95% confidence level.

Sample	Density (g/cm^3)	Error (g/cm^3)
**base**	2.64	0.01
**Na-deficient**	2.75	0.02
**Al-rich**	2.71	0.01

Supplementary Table 11. Densities (ρ), as measured by pycnometry, of the crystalline and amorphous pure ZIF-62 controls (1 and 30 min heat treatments). Error was calculated at 95% confidence level.

Sample	Density (g/cm^3)	Error (g/cm^3)
**ZIF-62**	1.47	0.08
**a_gZIF-62 – 1 min**	1.38	0.09
**a_gZIF-62 – 30 min**	1.42	0.04

Supplementary Table 12. Densities (ρ), as measured by pycnometry, of composites heat treated for 1 and 30 min. Error was calculated at 95% confidence level.

Sample	1 min		30 min	
	Density (g/cm^3)	Error (g/cm^3)	Density (g/cm^3)	Error (g/cm^3)
**$(a_g\text{ZIF-62})_{0.5}(\text{base})_{0.5}$**	1.60	0.1	1.84	0.3
**$(a_g\text{ZIF-62})_{0.5}(\text{Na-deficient})_{0.5}$**	1.69	0.3	1.83	0.1
**$(a_g\text{ZIF-62})_{0.5}(\text{Al-rich})_{0.5}$**	1.72	0.06	1.82	0.04

Supplementary References

1. Soper, A. K. & Barney, E. R. research papers Extracting the pair distribution function (PDF) from white beam x-ray total scattering data research papers. *J. Appl. Crystallogr.* **33**, 1–14 (2011).

2. Soper, A. K. GudrunN and GudrunX: Programs for Correcting Raw Neutron and X-ray Diffraction Data to Differential Scattering Cross Section. *Tech. Rep. RAL-TR-2011-013* (2011).

3. Keen, D. A. A comparison of various commonly used correlation functions for describing total scattering. *J. Appl. Crystallogr.* **34**, 172–177 (2001).

4. Prince, E. *International Tables for Crystallography. Vol. C, ch. 6.1,*. (International Union of Crystallography 2006, 2006). doi:https://doi.org/10.1107/97809553602060000600

5. Sławiński, W. A. Calculation of pair distribution functions for multiphase systems. *J. Appl. Crystallogr.* **51**, 919–923 (2018).

6. Soper, A. K. & Barney, E. R. Extracting the pair distribution function from white-beam X-ray total scattering data. *J. Appl. Crystallogr.* **44**, 714–726 (2011).

7. Banerjee, R. *et al.* High-throughput synthesis of zeolitic imidazolate frameworks and application to CO2 capture. *Science* **319**, 939–943 (2008).

8. Sigma-Aldrich. Imidazole sodium derivative technical grade. *Sigma-Aldrich* (2018). Available at: https://www.sigmaaldrich.com/catalog/product/aldrich/197637?lang=de&region=DE.

9. Lehnert, R. & Seel, F. Darstellung und Kristallstruktur des Mangan (II)- und Zink (II)-Derivates des Imidazols. *Zeitschrift für Anorg. und Allg. Chemie* **464**, 187–194 (1980).
